# Spatiotemporal transcriptomic map of glial cell response in a mouse model of acute brain ischemia

**DOI:** 10.1073/pnas.2404203121

**Published:** 2024-11-05

**Authors:** Daniel Zucha, Pavel Abaffy, Denisa Kirdajova, Daniel Jirak, Mikael Kubista, Miroslava Anderova, Lukas Valihrach

**Affiliations:** ^a^Laboratory of Gene Expression, Institute of Biotechnology of the Czech Academy of Sciences, Vestec 25250, Czech Republic; ^b^Department of Informatics and Chemistry, Faculty of Chemical Technology, University of Chemistry and Technology, Prague 16000, Czech Republic; ^c^Department of Cellular Neurophysiology, Institute of Experimental Medicine of the Czech Academy of Sciences, Prague 14220, Czech Republic; ^d^Department of Radiodiagnostic and Interventional Radiology, Institute of Clinical and Experimental Medicine, Prague 14021, Czech Republic; ^e^Faculty of Health Studies, Technical University of Liberec, Liberec 46001, Czech Republic

**Keywords:** ischemic stroke, single-cell transcriptomics, spatial transcriptomics, glia, neuroinflammation

## Abstract

The role of nonneuronal cells in the resolution of cerebral ischemia remains to be fully understood. To decode key molecular and cellular processes that occur after ischemia, we performed spatial and single-cell transcriptomic profiling of the male mouse brain during the first week of injury. Cortical gene expression was severely disrupted, defined by inflammation and cell death in the lesion core, and glial scar formation orchestrated by multiple cell types on the periphery. The glial scar was identified as a zone with intense cell–cell communication, with prominent ApoE-Trem2 signaling pathway modulating microglial activation. For each of the three major glial populations, an inflammatory-responsive state, resembling the reactive states observed in neurodegenerative contexts, was observed. The recovered spectrum of ischemia-induced oligodendrocyte states supports the emerging hypothesis that oligodendrocytes actively respond to and modulate the neuroinflammatory stimulus. The findings are further supported by analysis of other spatial transcriptomic datasets from different mouse models of ischemic brain injury. Collectively, we present a landmark transcriptomic dataset accompanied by interactive visualization that provides a comprehensive view of spatiotemporal organization of processes in the postischemic mouse brain.

## Significance

We performed spatial and single-cell transcriptomics on male mouse brains to map out the molecular and cellular processes governing the response to ischemic stroke during the first week after the injury. Focusing on glial cells, we documented their activation, the formation of the glial scar, and significant changes in cell–cell communication patterns. Interestingly, we identified various activated populations of oligodendrocytes that, while similar in their anti-inflammatory properties, differ in their immunogenic and metabolic characteristics. This challenges the long-held belief that oligodendrocytes play a passive role in neuropathologies. Altogether, we present a major contribution to the current understanding of the role of glial cells in stroke pathobiology, comprehensively mapping their acute response and role in the formation of the glial scar.

Ischemic stroke is an acute pathological condition caused by a sudden or gradual occlusion of cerebral arteries. The critical reduction in blood flow initiates a cascade of pathological events involving interactions between a large number of cell types (1, 2). The predominant pathophysiological process is neuronal death, accompanied by an extensive inflammatory response mediated by blood-borne immune cells and activated brain-resident cells (3). The activated resident glial cells exhibit a spectrum of pro- and anti-inflammatory properties, with their detrimental and beneficial roles largely determined through temporal and spatial factors, and interactions with other cell types (4). This creates a unique opportunity for the latest omics technologies and computational methods to provide a holistic understanding of the postischemic cellular mechanisms (5).

Recent single-cell transcriptomic studies have primarily focused on peripheral immune cells and microglia as the first responders to injury. Microglia have been observed to acquire four distinct states during early insult phases, characterized by increased phagocytosis (6–12), chemokine expression (6, 8, 11, 13), interferon response (6, 11–14), or proliferation (6, 11, 12, 15). Using spatial transcriptomics (ST), Han et al. (12) observed phagocytic microglia to reside in the proximal penumbra layer, while the interferon-responsive and proliferating formed a distal layer. Although astrocytes and oligodendrocyte-lineage cells contribute to forming the lesion-isolating glial scar (16), only few single-cell studies have profiled them in-depth. These studies observed that astrocytes and oligodendrocytes interact with microglia (17, 18), adopt an interferon-responsive state (8, 9), and participate in neurogenesis and synapse maintenance (14, 19, 20). Integrating single-cell and spatial transcriptomic methods, Scott et al. (21) annotated spatially distinct astrocyte populations differing in lipid shuttling. Although these pivotal studies provided important insights through integration of spatial location and transcriptional profiles, they investigated a single (8) or largely spread timepoints (17), missing the continuous development of ischemic injury.

Here, we examined the first 7 d following cortical ischemic stroke using spatial, single-cell, and bulk transcriptomics and immunohistochemistry. We identified key transcriptional programs and cell types responding to the injury, providing spatial and temporal context. We focused on glial cells in the ischemic penumbra, where they create and alter the glial scar through functional and compositional changes. Through our analysis, we highlighted the importance of Apoe signaling in microglial activation and the diversity of cellular states adopted by oligodendrocytes. We replicated these findings in two additional ST datasets employing different mouse models of ischemic injury. This resulting resource offers insights into the organization of cellular response in the early stroke pathology.

## Results

### Ischemic Brain Injury Severely Disrupts Cortical Gene Expression Landscape.

**Fig. 1.**
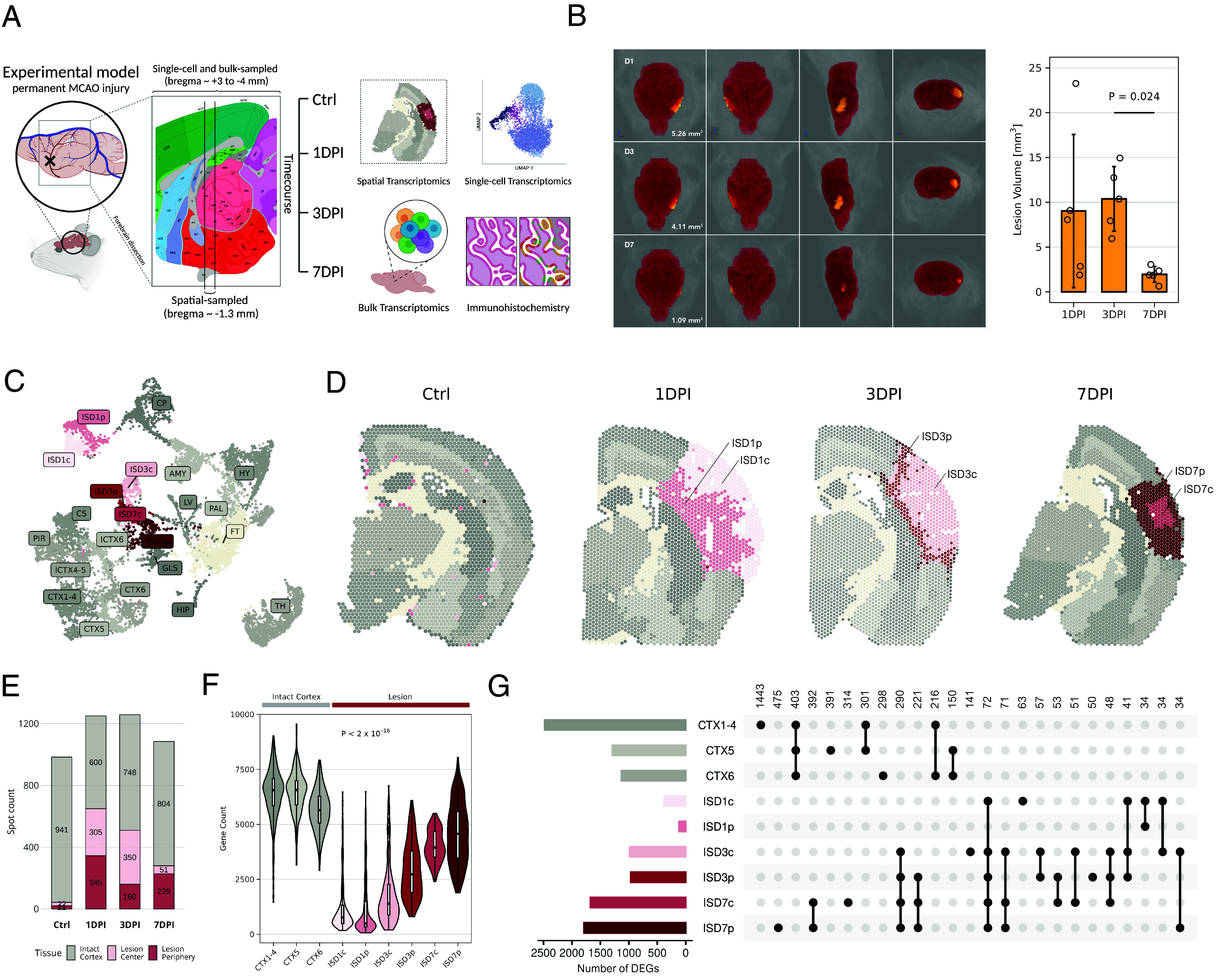
Spatial transcriptomics enable robust transcriptomic analysis of ischemia-induced injury. (*A*) Experimental design overview. (*B*) *Left*: 3D visualization of MCAO-injured mouse brain that underwent repeated MRI scanning at the three studied timepoints. The lesion is highlighted in yellow with volumes written out in the first panels, respectively. In the left-to-right order, the panels display the top–down, bottom–up, side, and front view. *Right*: Mean lesion volume as measured with MRI (n = 5 per timepoint). Error bars show SD. Wilcoxon’s rank sum test with Benjamini–Hochberg correction was used for statistical testing; the only significant difference is shown. (*C*) UMAP of spatial spots, color-coded by annotated brain regions (shades of gray for intact areas, red for lesions). Abbreviations: amygdala, AMY; caudoputamen, CP; cortical subplate, CS; cortex layers 1-4, CTX1-4; cortical layer 5, CTX5; lateral cortex layers 4-5, lCTX4-5; cortical layer 6, CTX6; lateral cortex layer 6, lCTX6; fiber tracts, FT; glia limitans superficialis, GLS; hippocampus, HIP; hypothalamus, HY; ischemic region day 1 center, ISD1c; ischemic region day 1 periphery, ISD1p; ischemic region day 3 center, ISD3c; ischemic region day 3 periphery, ISD3p; ischemic region day 7 center, ISD7c; ischemic region day 7 periphery, ISD7p; lateral ventricle, LV; pallidum, PAL; piriform area, PIR; thalamus, TH. (*D*) Spatial plot color-coded as in the previous panel. (*E*) Total spot counts of cortical areas. (*F*) Gene count in the areas of the intact cortex and lesions. Statistical significance of difference in gene count between the intact and lesioned area was assessed by an unpaired, two-tailed *t* test. (*G*) Overlaps of DEGs across intact and lesioned areas. Top 25 most numerous overlaps are shown with the overlap size written above.

To comprehensively view genome-wide expression changes postinjury, we performed a ST analysis in 3-mo-old male mice using the experimental model of permanent middle-cerebral artery occlusion (MCAO). The model mimics most clinical stroke cases, which do not receive timely treatment, leaving the clogged artery impassable (22, 23). We focused on the acute phase of the injury at four timepoints—no injury (ctrl), and day 1, 3, and 7 postinjury (DPI) (Fig. 1*A*).

To ensure we collect representative ischemic brain samples for ST, we first performed MRI on five mice per timepoint to assess the reproducibility of the MCAO injury (Fig. 1*B*). On average, the affected areas were similarly sized on days 1 and 3 (9.03 and 10.38 mm^3^, respectively) but significantly shrank on day 7 (1.95 mm^3^). Likewise, variability in size decreased over time (SD of ±8.55, 3.61, and 0.88 mm^3^, respectively), indicating injury stabilization. To monitor the progress at the level of an individual, we MRI-screened one additional MCAO-injured mouse at the three timepoints and confirmed the temporal lesion size retraction (Fig. 1*B*). Guided by these results, we collected representative coronal brain cryosections from bregma −1.3 ± 0.1 mm and processed them using the 10× Genomics Visium ST platform with the 55-μm spot resolution (*SI Appendix*, Fig. S1).

Using the Seurat ST pipeline, we first prepared a baseline characterization of the control section. The total area of 2,417 spots with a median of ~25,000 transcripts and ~6,000 genes per spot was mapped into 13 clusters corresponding to major brain regions of the Allen Brain Atlas (24) (*SI Appendix*, Fig. S2). For each region, we calculated differentially expressed genes (DEGs) and compared the top 100 DEGs with in situ hybridization (ISH) Allen Brain Atlas (Dataset S1). The comparison revealed significant overlap for all the major brain regions, and spatial expression patterns agreed visually in side-by-side comparisons (*SI Appendix*, Fig. S2). Together, this underscored the consistency of brain region identity at both the anatomical and transcriptional levels.

Integration and clustering with ischemic sections recapitulated brain anatomy and revealed lesion area diversification. Lesions primarily separated by time (ISD1, ISD3, and ISD7), and secondarily by central (c) or peripheral (p) location within the lesion (Fig. 1 *C* and *D*). Strict time-dependent clustering was interrupted by few ISD7p spots appearing at the day 3 lesion border, indicating gradual injury resolution from periphery to center. Notably, ischemia-affected clusters corresponded to brain areas supplied by the middle cerebral artery (25). Although cortical areas were similar in size among the sections (~1,000 spots), lesioned areas receded from 52 to 40% and ultimately 25% of the ST-profiled cortex, respectively (Fig. 1*E*). This agrees with the pilot MRI screens and highlights the correlation between the two independent technologies. Lesion dynamics were also reflected in the number of detected genes per spot, which progressively increased over time (Fig. 1*F*). Despite this increase, lesion gene counts remained lower than in the uninjured cortex, indicating incomplete recovery and changes in transcriptional programs and cellular composition.

To describe similarities and differences between individual timepoints and lesion locations, we computed DEGs (Dataset S2) and inspected their overlaps (Fig. 1*G*). Analysis revealed little to no direct reversion to the uninjured cortex state during the first week, despite increasing DEGs over time. Many DEGs were exclusive to specific timepoints or combinations of timepoints and locations, predominantly the 7DPI lesion. Approximately 40% of DEGs extended beyond a single timepoint, supporting the notion of gradual injury resolution from periphery to center (1, 16, 23, 26).

Collectively, the initial analysis revealed severe disruption in the cortical transcriptional profile due to ischemic injury, with a distinctive lesion core and an enveloping penumbra. Lesion dynamics were reflected in partially overlapping DEGs, highlighting the temporal and spatial nature of ischemia-induced processes.

### Ischemic Lesion Perturbs Cortical Processes and Cellular Composition.

**Fig. 2.**
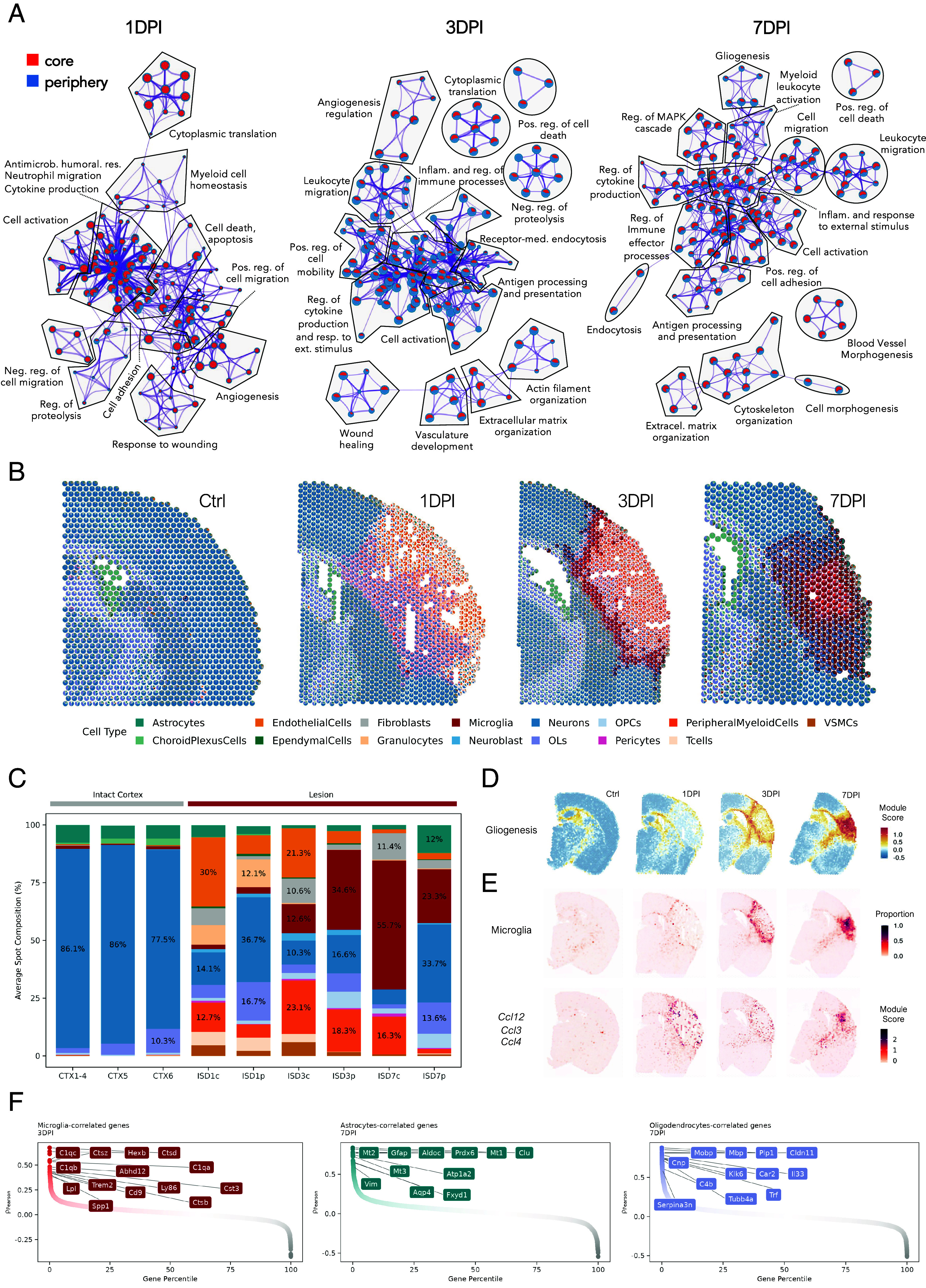
Ischemic injury involves a dynamic network of distinct BP and cell types. (*A*) GO network of enriched BP in the ST data, grouped by the parent GO term (Dataset S2). Each dot represents one enriched GO term, indicating the number of contributing DEGs (dot size) and core–periphery contribution (red-blue color scale, respectively). Prepared with Metascape (32). (*B*) Spatial plot of deconvoluted cell type proportions per spot. (*C*) Mean cell type proportion per brain region estimated by RCTD (33) using Zeng et al., as a ref. 10. Color-coding matches the previous panel. (*D*) Projection of gliogenesis module score for Gliogenesis GO term (GO:0042063). (*E*) Projection of predicted microglial proportion (*Top*) and module score of CC chemokine expression (*Ccl12, Ccl3, Ccl4*; *Bottom*) per spot. (*F*) Pearson correlations between genes and cell type proportions in selected timepoints. Selected highly correlating genes are highlighted.

To characterize ischemic injury functionally over time and space, we performed enrichment analysis on lesion DEGs (Dataset S2) and visualized up-regulated biological processes (BP) in timepoint-specific networks, highlighting contributions from central and peripheral areas (Fig. 2*A*). On day 1, processes associated with leukocyte migration, adhesion, cytokine production, cell activation, and response to wounding were prominent, reflecting increased infiltration and activation of peripheral immune cells (1, 3, 27). Additionally, terms related to cell death, apoptosis, proteolysis, extracellular matrix organization, and angiogenesis suggested an early initiation of reparative processes (28, 29) (*SI Appendix*, Fig. S3). Most processes were primarily activated in the lesion center, likely due to milder or delayed damage in the periphery (23). This is consistent with the gradient-like character of the ISD1 peripheral cluster, which contrasts the enveloping-like shape observed at later timepoints. Processes initiated on day 1 persisted on day 3, often to a higher degree (Fig. 2*A* and Dataset S2), with reparative machinery prominent in the center and inflammation primarily in the periphery, involving immune cell activation and glial cell migration (4, 16) (*SI Appendix*, Fig. S3). By day 7, most processes, including inflammation, were relocalized to the lesion center. Gliogenesis, axon ensheathment, and synapse pruning were more pronounced in the periphery, likely indicating the first signs of neural reorganization (26, 30, 31).

To complement this analysis with cell-type-specific context, we used RCTD algorithm (33) to deconvolute cell-type proportions within spots. For the reference, we selected a single-cell stroke study by Zeng et al. (10) due to its matching experimental setup and comprehensive coverage of 15 cell types, both elements crucial to the deconvolution quality (34). The injury dramatically changed cortical cellular composition (Fig. 2*B*). Lesioned areas showed significant neuron reduction, counteracted by time- and space-distinctive accumulation of glia, peripheral immune cells, fibroblasts, and vascular cells (Fig. 2*C* and *SI Appendix*, Fig. S3). On day 1, neuronal content decreased progressively from center to periphery, indicating gradual injury expansion (23). The near-complete loss of neurons on day 3 improved by day 7, with neuronal content increasing in the periphery. While peripheral immune cells were absent in the uninjured cortex, granulocytes, peripheral myeloid cells, and T cells infiltrated the lesion on day 1. In later timepoints, their numbers receded almost exclusively to peripheral myeloid cells in the lesion center. Vascular cells and fibroblasts, involved in angiogenesis and fibrotic scar formation, displayed distinct temporal and spatial profiles. Both preferentially localized in the center, with vascular cells present until day 3, and fibroblasts across all timepoints.

Deconvolution analysis also captured prominent glial proliferation and differentiation encompassing astrocytes, oligodendrocytes, and microglia (Fig. 2*D* and *SI Appendix*, Fig. S3). On day 1, oligodendrocytes increased proportionally in the heavily injured lesion periphery, with the lesion boundary delineated by microglia. Microglia colocalized with several CC-family chemokines (*Ccl3, Ccl4, Ccl12*, Fig. 2*E*), suggesting their contribution in attracting and activating infiltrating peripheral immune cells and other glia (35–38). On day 3, myeloid and endothelial cells dominated the lesion composition. Correlation with markers of microglial activation, such as inflammatory signaling molecules (*C1qa/b/c, Spp1*), lipid metabolism genes (*Abhd12, Lpl*), cathepsins (*Ctsb, Ctsd*), and their inhibitors cystatins (*Cst3, Cst7*), along with a thicker microglia-rich layer of spots, indicated forming glial scar around the lesion center (16, 39, 40) (Fig. 2*F* and Dataset S3). In support, microglia were joined by oligodendrocytes and oligodendrocyte precursor cells, which we validated by immunostaining PGDFRα^+^ in OPCs, PLP1^+^ in oligodendrocytes, and IBA1^+^ in microglia (*SI Appendix*, Fig. S4). By day 7, the receding lesion was largely populated by myeloid cells, with an increasing lesion periphery composed of astrocytes and oligodendrocytes. Interestingly, these glial populations correlated well with glial markers identified in various neuropathologic conditions (41–45), such as *Gfap, Vim, Mt1* in astrocytes, and *Serpina3n, C4b, Klk6* in oligodendrocytes (Fig. 2*F* and Dataset S3).

Altogether, the functional and cellular characterization of the ischemic lesion revealed a profound inflammatory response with distinct spatial localization over time. This response was accompanied by changes in cell type composition, with initial contributions from peripheral immune and vascular cells, later succeeded by glia and fibroblasts. Glial cells gradually became activated, forming a glial scar that encloses the ischemic core.

### Cell–Cell Communication Analysis Identifies Increased Glia-Oriented Crosstalk in the Lesion Periphery.

Next, we applied the SpaTalk algorithm (46) to decompose deconvoluted spatial spots into individual single cells, inferring plausible ligand–receptor (LR) interactions between spatially proximal pairs of cells. We focused on interactions in the lesion periphery to elucidate the coordination involved in glial scar formation.

**Fig. 3.**
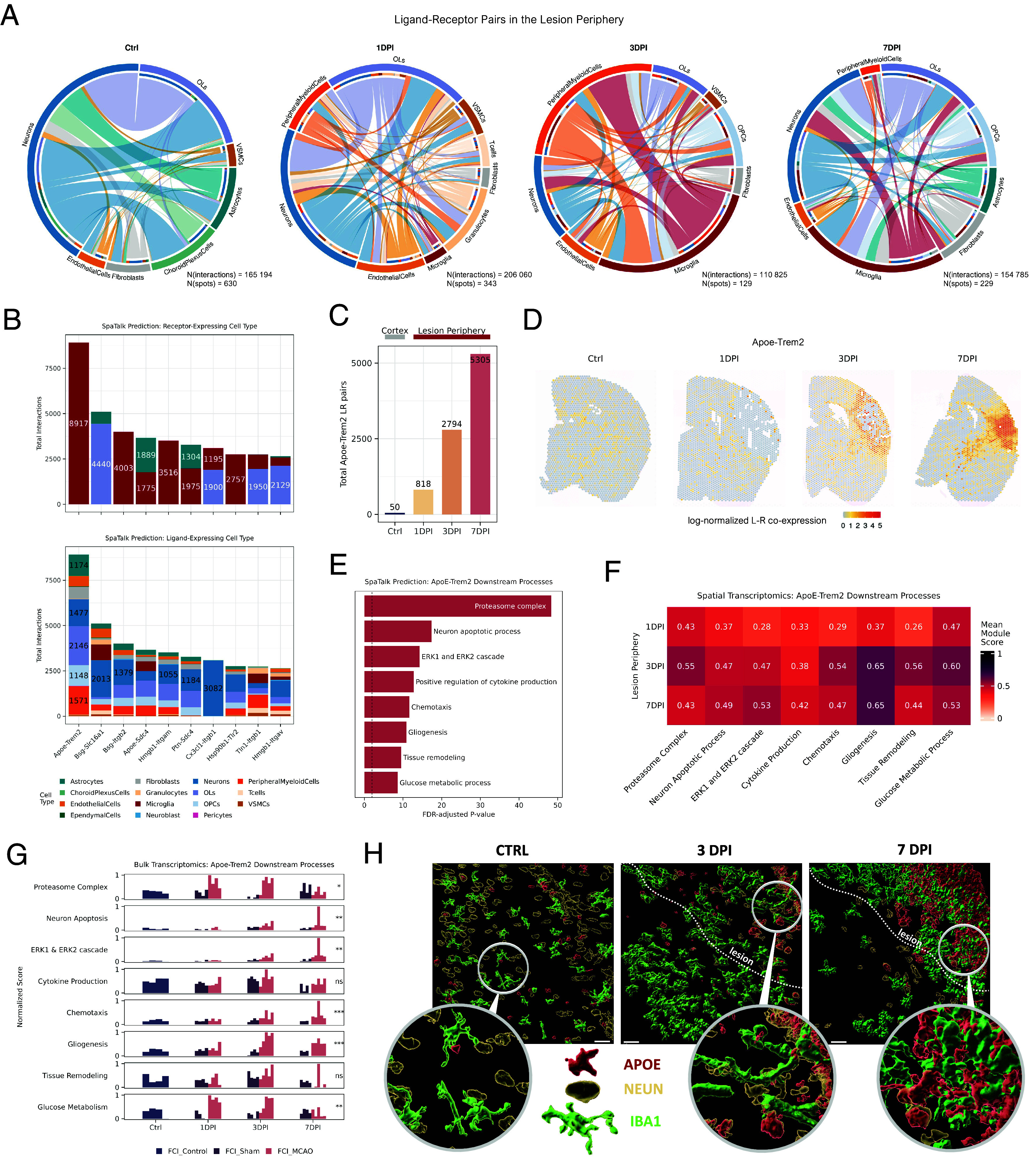
Apoe-Trem2 is an important constituent of postischemic gliosis in the lesion periphery. (*A*) Summary of ligand–receptor interactions in the lesion periphery in respective timepoints with total numbers of estimated interactions and spatial spots next to each plot. For the control condition, an entire uninjured cortex was used. (*B*) The summary of top 10 most numerous ligand–receptor pairs with glia as receivers in the lesion periphery cumulatively across the studied timepoints. Upper panel: color-coded glial cells as receptors. *Bottom* panel: color-coded ligand-expressing cell types. (*C*) Total number of estimated *Apoe-Trem2* pairs per timepoint. (*D*) Spatial organization of *Apoe-Trem2* coexpression. (*E*) Gene-ontology enrichment of the predicted *Apoe-Trem2*-induced genes. (*F*) Module score of the enriched *Apoe-Trem2*-induced GO processes in the respective lesion peripheries in the ST data. (*G*) Normalized score for *Apoe-Trem2*-induced processes in bulk transcriptomic data (minimum of n = 4 replicates per timepoint). The Wilcoxon rank sum test with Benjamini–Hochberg correction was used to test the significance in score difference between non-MCAO (control and shams) and MCAO samples (*ns* = nonsignificant, *P < 0.05, **P < 0.01, ***P < 0.001). (*H*) 3D reconstruction of microglia (IBA1), neuron cell bodies (NEUN), and APOE networks using immunohistochemistry in respective timepoints. (Scale bars, 20 μm.)

The analysis revealed a dramatic shift in the interactome landscape, moving from neurons and oligodendrocytes to vascular, immune, and glial cells (Fig. 3*A*). For instance, neuronal *Bsg*, crucial for neuronal metabolism under homeostatic conditions (47), increasingly targeted adhesion receptors of immune cells in early lesions (Dataset S4). *Bsg*’s role in reducing extracellular matrix density (47) suggests it may fine-tune the localization and activity of the infiltrating immune cells. Examining LR pairs where glia acted as communication receivers, we observed significant shifts from day 3 (Dataset S4). Microglia, due to their increasing proportions, became more frequent signal senders and receivers (Fig. 3*A*), underscoring their importance in glial scar formation (48). Their integrin receptors *Itgb1, Itgb2, Itgam* were among the most targeted, with interactions involving cellular adhesion (*Tln1, Jam3, Spp1*), motility (*Hmgb1, Cx3cl1*), and innate immune responses (*Tgfb1, Cd14, Lgals3, Lgals3bp*) (Fig. 3*B* and Dataset S4). Microglia not only received signals but also actively instructed other cell types, particularly oligodendrocytes, to enhance adhesion (*Tgfb1-Itgb1*, *Lgals3bp-Itgb1*) or lactate release (*Bsg-Slc16a1*), possibly aiding in microglial metabolic reprogramming (49).

Despite the pronounced integrin-oriented LR communication, the dominant interaction in the lesion periphery was Apolipoprotein E and Triggering Receptor Expressed on Myeloid cells 2 (*Apoe-Trem2*; Fig. 3*B*). *Apoe-Trem2* is well studied in various neuropathologies (50). *Apoe*, a lipid-transporting protein, colocalizes with lesions and modulates microglial activation through *Trem2* signaling. Our analysis estimated a more than 100-fold increase in microglia-directed *Apoe-Trem2* pairs from the control cortex to lesion periphery on day 7 (Fig. 3*C*). Moreover, *Apoe-Trem2* coexpression was observed in the lesion-proximate cortex and fiber tracts (Fig. 3*D*), indicating secondary microglial activation due to damaged axonal connections (51). Microglial response to *Apoe-Trem2* is multifaceted (50). To delineate direct *Apoe-Trem2* effects, we gathered genes within its LR-targeted signaling networks and enriched them for gene ontology (GO) terms (Fig. 3*E*). *Apoe-Trem2*-enriched processes in microglia were linked to cellular activation (ERK1/2 cascade, glucose metabolism, cytokine production), debris clearance (proteasome complex, neuron apoptosis), cellular morphology (gliogenesis, tissue remodeling), and chemoattraction (chemotaxis, cytokine production). Visualizing these processes in ST sections showed upregulation in the lesion, varying in intensity across timepoints (Fig. 3*F* and *SI Appendix*, Fig. S5). On day 1, these processes were least pronounced but delineated the lesioned cortex (*SI Appendix*, Fig. S5). By day 3, their presence intensified, localizing more to the lesion periphery in line with *Apoe-Trem2* (*SI Appendix*, Fig. S5). By day 7, activity differences between the periphery and core largely diminished.

To verify our observations, we replicated the experiment using bulk RNA-sequencing and immunostaining, with a minimum of n = 4 and 3 mice per condition, respectively. Bulk data inspection of *Apoe-Trem2* coexpression and its downstream processes confirmed temporal trends seen in the ST data (Fig. 3*G* and *SI Appendix*, Fig. S5). Early elevated activity of proteasome complexes and glucose metabolism persisted until day 3, followed by a peak in tissue remodeling and cytokine production on day 3, alongside increased ERK1/2 cascade and gliogenesis activity. Immunohistochemistry revealed significant microglial activation, with microglia tripling their volumes compared to controls (*SI Appendix*, Fig. S5). Increased microglial density and volume coincided with elevated *Apoe* levels in the lesion (Fig. 3*H*), corroborating the accumulation seen in ST data. Furthermore, deconvolution of bulk data using Zeng et al. as a ref. 10 validated the ST data deconvolution. Despite using nearly an entire ipsilesional hemisphere for bulk samples, the correlation with ST proportions was moderately high (concordance correlation coefficient = 0.879, *SI Appendix*, Fig. S5). The least concordant were vasculature-related cells (~1% in proportions), while more abundant neurons and glia showed good agreement. Statistical testing of bulk cell type proportions confirmed a significant decline in neurons and increase in microglia over the studied period (*SI Appendix*, Fig. S5).

In summary, SpaTalk analysis revealed critical ligand–receptor interactions for glial scar formation in the lesion periphery, dominated by *Apoe-Trem2*, highlighting its role in modulating microglial activation.

### Single-Nucleus Profiling of Postischemic Brain Documents the Rise of Reactive Glia.

Previous analyses evidenced presence of reactive glia in the ischemic brain. To characterize them, we performed single-nucleus RNA-seq (snRNA-seq) of control and postischemic brains on days 1, 3, and 7. We sampled tissue from +3 to −4 mm from bregma, comparable to the ST (Fig. 1*A*). We obtained 7,946 high-quality nuclei, classified into 11 major cell type populations, including glutamatergic and GABAergic neurons, neuroblasts, and ependymal, vascular, and glial cells (*SI Appendix*, Fig. S6). We focused on three glial populations: astroependymal cells (n = 763), microglia (n = 426), and oligodendrocyte lineage cells (n = 1,433) and analyzed them in detail.

**Fig. 4.**
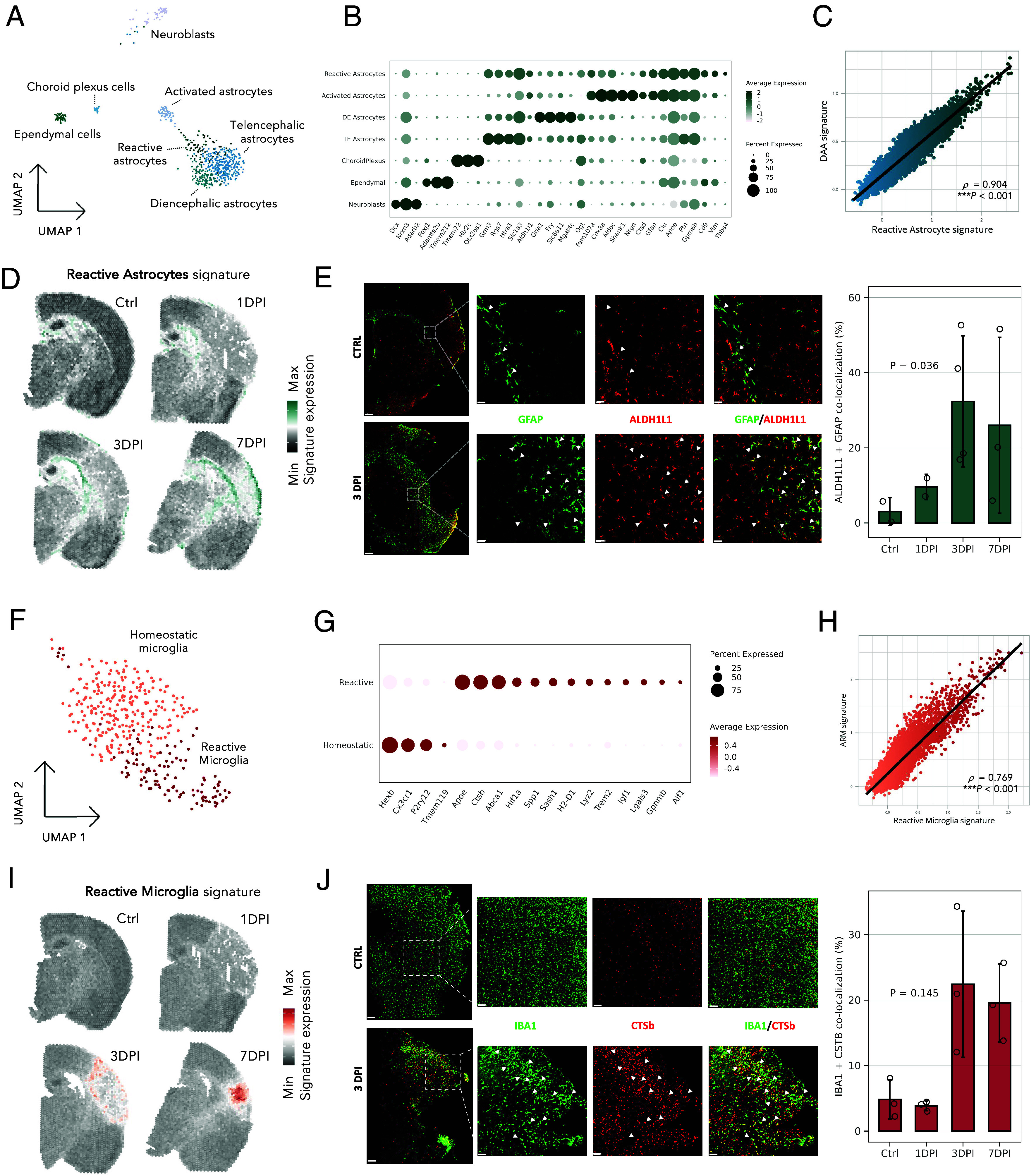
Glia acquire reactive states in response to the post-MCAO conditions. (*A*) UMAP of astroependymal populations (n = 610 nuclei). (*B*) Marker genes of astroependymal populations. Expression level (color scale) and the percentage of population expressing the marker (dot size) are shown. (*C*) Correlation between the expression signatures of the reactive astrocytes (*x* axis) and AD-associated DAAs (45) (*y* axis) in the ST dataset, measured per spot and quantified with Spearman’s rank correlation coefficient *ρ*. (*D*) Projection of reactive astrocytes expression signature. (*E*) Coronal mouse brain sections immunostained for astrocytic canonical (ALDH1L1) and reactivity marker (GFAP). (Scale bars of 200 μm and 20 μm for the hemisphere and the *Inset* image, respectively.) Signal colocalization was calculated using a minimum of two sections per mouse, with a minimum of two mice per condition. Error bars show SD. Statistical significance between control and injured samples was assessed using the unpaired two-sided Wilcoxon rank sum test. (*F*) UMAP of microglial populations (n = 362 nuclei). (*G*) Marker genes of microglial populations. Expression level (color scale) and the percentage of populations expressing the marker (dot size) are shown. (*H*) Correlation between the expression signatures of reactive microglia (*x* axis) and AD-associated ARMs (43) (*y* axis) in the ST dataset, measured per spot and quantified with Spearman’s rank correlation coefficient *ρ*. (*I*) Projection of reactive microglia expression signature. (*J*) Coronal mouse brain sections immunostained for microglial canonical (IBA1) and reactivity marker (CTSB). (Scale bars of 200 μm and 20 μm for the hemisphere and the *Inset* image, respectively.) Signal colocalization was calculated on minimum of two sections per mouse, with a minimum of three mice per condition. Error bars show SD. Statistical significance between control and injured samples was assessed using the unpaired two-sided Wilcoxon rank sum test.

In the astroependymal cluster, we identified eight populations: neuroblasts (*Dcx*), choroid plexus cells (*Tmem72*), ependymal cells (*Foxj1*), and four astrocyte populations (Fig. 4*A* and Dataset S5). Consistent with Zeisel et al. (52), two astrocyte populations were homeostatic (*Slc1a3*), with region-distinctive origins in the telencephalon (*Grm3*) and diencephalon (*Gria1*s) (Fig. 4*B* and *SI Appendix*, Fig. S5). Another population, activated astrocytes, was characterized by high levels of ribosomal and mitochondrial reads (*SI Appendix*, Fig. S6), activated energy metabolism (*Cox8a*, *Aldoc*), and genes involved in neuron maintenance and survival (*Shank1, Nrgn*; Dataset S5). Combining key marker expressions into an expression module, the activated astrocytes’ signature was enriched in the hippocampal dentate gyrus, caudoputamen, and subpial regions (*SI Appendix*, Fig. S6). The localization and expression signature indicate a maturing population (53), possibly with a neurogenic potential (54). The fourth astrocyte population showed a reactive status, having elevated expression of reactivity (*Gfap*, *Apoe, Clu*), cell adhesion (*Cd9, Ptn*), proliferation (*Vim, Thbs4*), lysosomal activity (*Ctsd*), and membrane lipid rafts (*Gpm6b*) genes (*SI Appendix*, Fig. S6 and Dataset S5). Comparing their markers with disease-associated astrocytes (DAAs) identified in Alzheimer’s disease (AD) (45), we found significant overlap (*SI Appendix*, Fig. S6) and strong correlation in the ST data (Spearman’s *ρ* = 0.904, ***P < 0.001 Fig. 4 *C* and *D*), suggesting similar mechanisms are activated in postischemic and AD astrocytes. Immunostaining confirmed localization of ALDH1L1+GFAP+ astrocytes in the lesion periphery and their enrichment in postischemic conditions (Fig. 4*E*).

Microglia divided into homeostatic (*P2ry12*) and reactive (*Apoe, Spp1*) populations (Fig. 4*F*). Reactive microglia overexpressed genes related to cell migration, proliferation, adhesion (*Gpnmb, Sash1, Lgals3*), protein secretion (*Spp1, Igf1*), tissue remodeling (*Hif1a*), and lipid transport (*Abca1*, *SI Appendix*, Fig. S6 and Dataset S5). Reactive microglia peaked on day 3, comprising almost half of the collected microglia (*SI Appendix*, Fig. S6). Comparing their signature with AD-associated microglia, particularly the activated response microglia (ARM) (43), we found significant overlap (*SI Appendix*, Fig. S6) and strong correlation in the ST data (Spearman’s *ρ* = 0.769, ***P < 0.001, Fig. 4*H*), indicating activation of similar transcriptional programs in acute and neurodegenerative pathology. Projecting their signature into ST documented microglia accumulating on the lesion periphery on days 1 and 3, then expanding to the lesion center (Fig. 4*I*). Immunostaining for IBA1 and reactive CTSb markers confirmed IBA1+CSTb+ microglia in the lesion periphery and their enrichment postischemia (Fig. 4*J*).

Oligodendrocyte lineage cells were annotated using Zeisel et al.’s standardized nomenclature (52). We identified four populations: oligodendrocyte precursor cells (OPC; *Pdgfra, Sox6*), newly formed oligodendrocytes (NFOL; *Tcf7l2*, *Prom1*, *Nckap5*), a major population of mature oligodendrocytes (MOL; *Plp1, Grm3, Mast4*), and a MOL population of reactive oligodendrocytes (*Serpina3n, C4b*) (*SI Appendix*, Fig. S7). Reactive oligodendrocytes were exclusive to ischemic samples, overexpressed long-noncoding RNAs (*Neat1, Pvt1*), and genes related to cell motility, polarity (*Rhoj*, *Fmnl2*), division (*Pkp4, Cdc14a*), and immune response regulation (*Il33, C4b, Gab2*) (Dataset S5). Interestingly, similar profiles were previously documented in neurodegenerative contexts (41, 44). In the spatial dataset, reactive oligodendrocytes’ signature was detected in oligodendrocyte-rich white matter tracts in both control and ischemic sections, with signal strengthening and encircling the lesion (*SI Appendix*, Fig. S7).

To summarize, snRNA-seq confirmed the emergence of reactive glial populations postischemia. Comparing them with AD-associated reactive glia, they up-regulated similar pathways and immunostaining validated their localization in lesion-respective areas.

### A Spectrum of Reactive Oligodendrocytes Emerges After Ischemic Brain Injury.

**Fig. 5.**
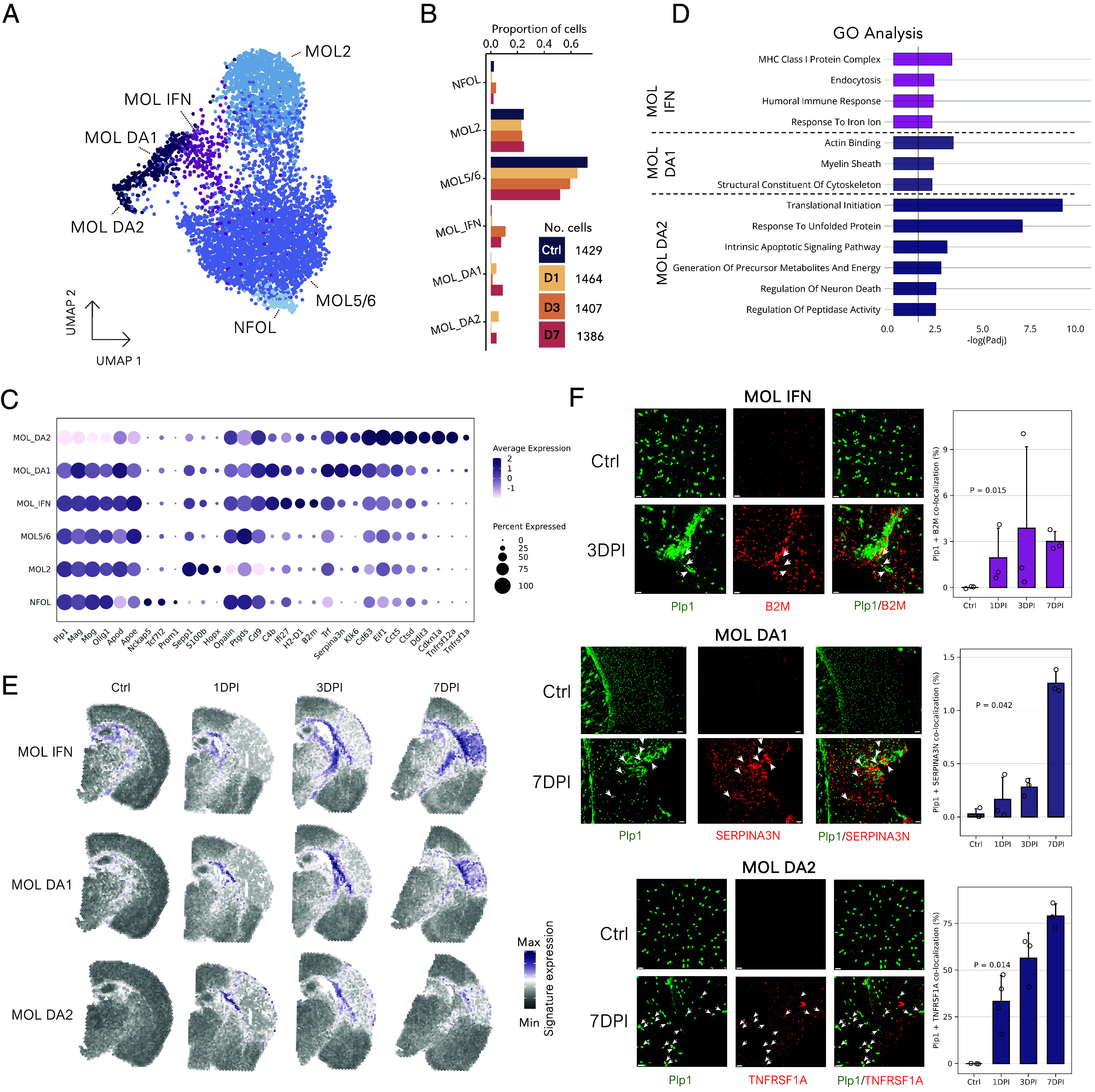
Ischemic conditions induce a spectrum of reactive oligodendrocyte populations. (*A*) UMAP of populations in the integrated dataset of single nuclei (n = 1,113) and single cells (n = 4,673) of post-MCAO oligodendrocytes. Abbreviations: Newly formed oligodendrocytes, NFOL; mature oligodendrocytes type 2, MOL2; mature oligodendrocyte types 5 and 6, MOL5/6; interferon-responsive mature oligodendrocytes, MOL IFN; disease-associated mature oligodendrocytes 1, MOL DA1; disease-associated mature oligodendrocytes 2, MOL DA2. (*B*) Proportions of oligodendrocyte populations at the respective timepoints. (*C*) Marker genes of oligodendrocyte populations. Expression level (color scale) and the percentage of populations expressing the marker (dot size) are shown. (*D*) Representative enriched GO terms for trauma-associated oligodendrocytes, categorized as ontology terms belonging to BP, CC, or molecular function (MF). FDR-adjusted *P* value is shown. (*E*) Projections of reactive oligodendrocyte expression signatures. (*F*) Immunohistochemistry validation. Coronal mouse brain sections stained for canonical oligodendrocyte marker (*Plp1*) and reactive oligodendrocyte populations: B2M for MOL IFN, SERPINA3N for MOL DA1, and TNFRSF1A for MOL DA2, respectively. Colocalization in a minimum of three sections per mouse, with a minimum of three mice per condition. Error bars show the SD. (Scale bars of 20 μm for B2M and TNFRSF1A and 50 μm for SERPINA3N images, respectively.) Statistical significance between control and injured samples was assessed using the unpaired two-sided Wilcoxon rank sum test.

Prompted by evidence of the immunomodulatory role of OLs in neurodegeneration (41, 44), we opted to further characterize OLs in ischemia. Using the same experimental design, we FACS-enriched *Plp1+* cells from control and ischemic brains and performed single-cell RNA-seq (scRNA-seq). After quality control, we identified 4,573 high-quality OLs and integrated them with single-nucleus OLs (*SI Appendix*, Fig. S7). The integrated dataset distinguished two mature OL populations, MOL2 (*S100b, Hopx*) and MOL5/6 (*Ptgds, Opalin*), improved NFOL annotation (*Tcf7l2*, *Prom1*), and recovered three pathology-enriched MOL populations (Fig. 5 *A* and *B*). Following Pandey et al.’s annotation (41), we named them interferon-responsive, and disease-associated mature OLs 1 and 2 (MOL IFN, MOL DA1, and MOL DA2).

The MOL IFN population was characterized by terms related to interferon response (*Ifi27, Ifi27l2a*) and antigen presentation by MHC class I (*H2-D1, B2m*; Fig. 5 *C* and *D*). This population also up-regulated genes associated with endocytosis (*Trf, Gsn*) and cell adhesion (*Cd9*, *Cldn11*), indicating an active injury response. Their spatial expression progressively strengthened (Fig. 5*E*), expanding from the lateral ventricle along the lesion periphery to the corpus callosum and deeper fiber tracts. On day 7, the signature encircled the lesion core, suggesting crosstalk with other cells forming the glial scar. MOL DA1 had elevated ribosomal RNA content (*SI Appendix*, Fig. S7) and highest expression levels of reactive marker genes—*Serpina3n, C4b,* and *Klk6* (44) (Fig. 5*C*). GO analysis indicated roles in myelination and cytoskeletal restructuring (*Arpc1b, Tubb3*; Fig. 5*D*). Additional markers indicated regulation of extracellular serine protease activity (*Serpina3n, Klk6*), cell adhesion (*Cd9, Cd63*), metal ion homeostasis (*S100a6, S100a16, Calm2, Trf*), and enhanced lipid metabolism (*Apod, Fabp5*). Spatially, MOL DA1 signature concentrated in the corpus callosum on the lesion periphery, expanding on day 7 to encircle the lesion (Fig. 5*E*). In contrast to the MOL IFN signature, it was reduced in deeper white matter tracts. MOL DA2, the most distinct population, had proportionally high ribosomal content and up-regulated genes associated with translation initiation (*Eif1, Eif4a1*) and protein-folding chaperons (*Cct2, Cct5*; Fig. 5 *C* and *D* and *SI Appendix*, Fig. S7). Their activated profile also included metabolic processes (*Cox5a, Atp5a1*) and regulation of peptidase activity (*Serpina3n, Ctsb, Ctsd, Timp1*). They expressed genes related to stress (*Fos, Jun*), cell-cycle regulation (*Cdkn1a*), and apoptosis (*Gadd45b, Tnfrsf12a, Tnfrsf1a, Ddit3*), indicating a struggle for survival. Notably, myelinating genes (*Plp1, Mog, Mag*) were down-regulated, suggesting reduced myelination capacity. The spatial expression of MOL DA2 was prominent on day 1 in the corpus callosum near the lateral ventricle, decreasing over time but remaining in the outermost OL layer surrounding the lesion (Fig. 5*E*).

Marker genes and functional annotations of pathology-enriched populations showed substantial similarity to those in neurodegenerative contexts. All three reactive populations were enriched for the signature of AD-associated DOLs (44), and the three distinctive OL populations shared across models of AD and multiple sclerosis (41) (*SI Appendix*, Fig. S7). To validate these populations, we costained coronal mouse brain sections for canonical *Plp1* with B2M for MOL IFN, SERPINA3N for MOL DA1, and TNFRSF1A for MOL DA2 (Fig. 5*F*). For each pair, double-positive cells were minimal in control brains but increased progressively, peaking on day 7. In accordance with ST, the colocalization signal accumulated in the lesion periphery, the area of glial scar formation.

In conclusion, combining snRNA-seq and scRNA-seq identified a spectrum of reactive oligodendrocyte populations postischemia. Despite expressing common markers like *Serpina3n* or *C4b* reflecting their potential to reduce neuroinflammation (55, 56), these populations displayed unique immunogenic and metabolic gene profiles. They also exhibited specific spatiotemporal organization and similarities to reactive oligodendrocytes in neurodegenerative diseases.

### Glial Prominence in the Ischemic Lesion Periphery Is Recapitulated in Published ST Datasets.

**Fig. 6.**
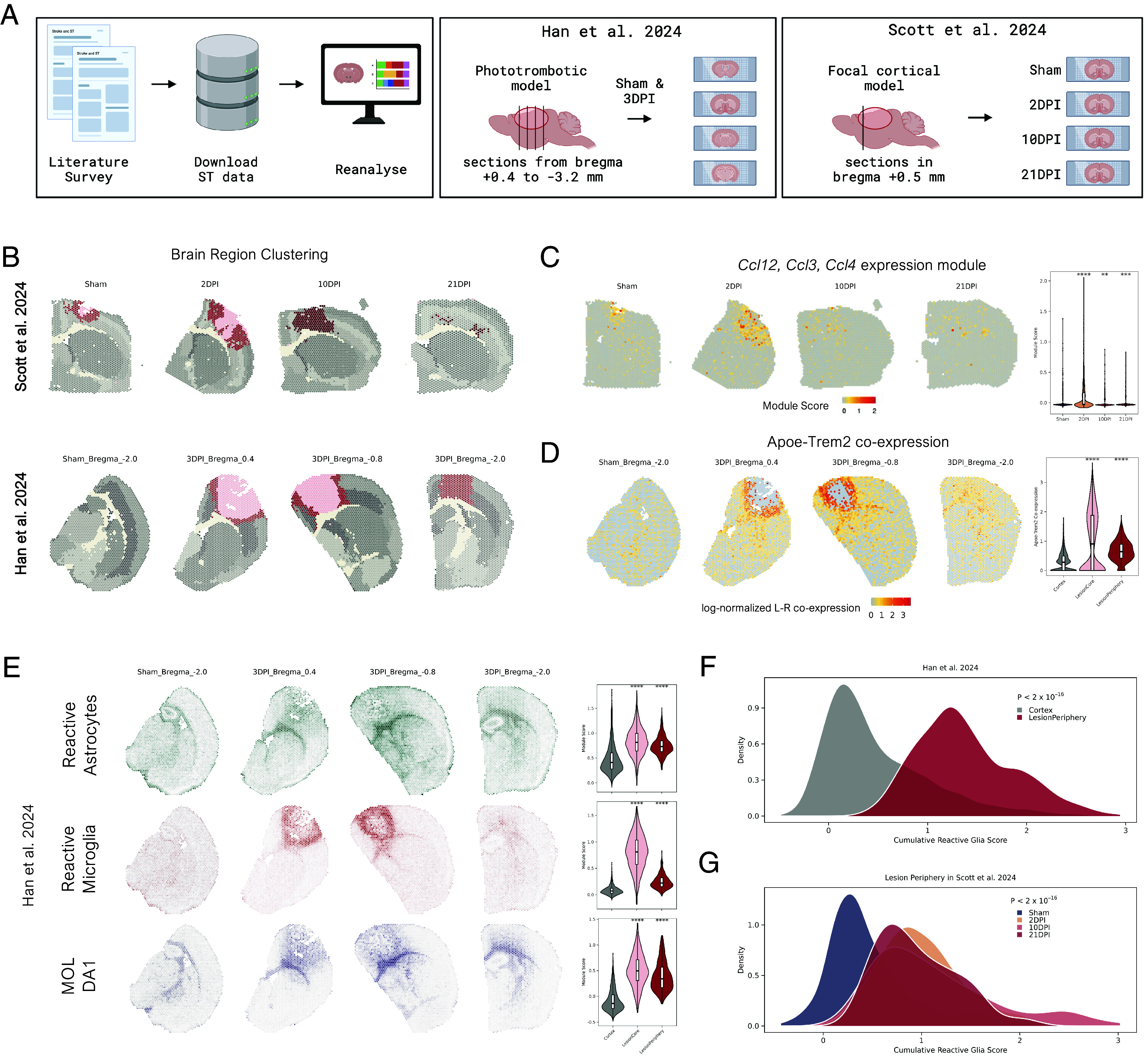
Reanalysis of public ischemia-related ST datasets. (*A*) Graphical summary of selected publicly available ST stroke datasets. (*B*) Spatial plot color-coded for brain regions matching that in Fig. 1*D* for two reanalyzed datasets. The uninjured areas are displayed in shades of gray, lesioned in the shades of red. (*C*) Projection of module score of CC chemokine expression (*Ccl12, Ccl3, Ccl4*) per spot in sections from Scott et al. (21). Absolute values are shown in the right-side violin plot. The statistical difference was assessed with an unpaired two-sided *t* test. (*D*) Spatial organization of *Apoe-Trem2* coexpression in sections from Han et al. (12), with absolute values shown in the *Right*-side violin plot. The statistical difference was assessed with an unpaired two-sided *t* test. (*E*) Projections of reactive glial expression signatures in Han et al. (12). (*F*) Comparison in Cumulative Reactive Glia Score, a sum of signatures plotted in panel *E*, for spots of the uninjured cortex with lesion periphery in the visualized sections of Han et al. (12). Statistical significance was assessed by an unpaired, two-tailed *t* test. (*G*) Comparison in Cumulative Reactive Glia Score between the uninjured cortex (Sham) and lesion peripheries’ respective timepoints in the sections of Scott et al. (21). Statistical significance was assessed by one-way ANOVA.

To validate the principal biological themes of our study, we reviewed additional ischemia-related ST datasets (Fig. 6*A*). We identified two relevant studies: Han et al. (12), employing a photothrombotic mouse model sectioned at evenly spaced intervals from bregma +0.4 to −3.2 mm at a single timepoint 3DPI, and Scott et al. (21), employing a focal cortical mouse model sectioned at bregma +0.5 mm at 2, 10, and 21DPI. Reanalyzing these datasets contextualized our findings across the acute lesion, extended the time window to later timepoints, and compared results in different ischemia models.

For both datasets, clustering analysis reproduced the severe disruption of the cortical landscape postischemia. In Han et al., the lesion spanned multiple sections, separating it into the core and periphery over a minimal distance of 1.2 mm (Fig. 6*B*). Similarly, in Scott et al., the earliest timepoint showed lesion separation into core and periphery (Fig. 6*B*). In both studies, reduced gene counts in lesion cores were a major differentiating factor, as observed in our study (Fig. 1*F* and *SI Appendix*, Fig. S8). Furthermore, in Scott et al., with a fixed location, the lesion progressively shrank, transforming to have a dominating periphery over time. While this may be partly attributed to lesion variability, it generally recapitulated our ST and MRI observations (Fig. 1 *B*–*E*).

Next, we investigated the temporal and spatial localization of expression patterns associated with glial accumulation in the lesion periphery. Using identical approaches, we computed expression enrichment for the *Ccl12, Ccl3,* and *Ccl4* chemokine module (Fig. 6*C*) and ligand–receptor interaction of *Apoe-Trem2* (Fig. 6*D*). The chemokine module was concentrated in lesioned areas, mainly at earlier timepoints (Fig. 6*C*), located near the lesion core–periphery boundary (*SI Appendix*, Fig. S8). This reproduced our observation that chemokine expression delineates the lesion boundary early postinjury (Fig. 2*E*). In both reanalyzed studies, *Apoe-Trem2* was significantly up-regulated in lesions, with a spatial gradient increasing toward the core–periphery boundary (Fig. 6*C* and *SI Appendix*, Fig. S8). Similar to our data (Fig. 3*D*), *Apoe-Trem2* was less distinct within the lesion 1 wk postinjury and later (*SI Appendix*, Fig. S8).

Last, we investigated whether reactive glial expression signatures identified in our experiments (Figs. 4 and 5) were similarly elevated and localized in reanalyzed studies. With identical computational settings, we observed significant enrichment of reactive signatures for all three glial populations in lesion areas compared to the uninjured cortex (Fig. 6*E* and *SI Appendix*, Fig. S8). The microglial reactive signature formed the inner layer of the glial scar at the core–periphery border. Reactive astrocyte and oligodendrocyte signatures accumulated outside the microglial layer, showing a receding gradient in the corpus callosum-cortex orientation. To summarize the enrichment of these signatures, we introduced a Cumulative Reactive Glia Score by summing their signature values. In Han et al., the comparison between the uninjured cortex and lesion periphery revealed a prominent difference (Fig. 6*F*), supporting glial scar formation. In Scott et al., statistical testing confirmed the shift toward reactive glia expression in lesioned areas, with comparable scores between timepoints (Fig. 6*G*).

Taken together, reanalysis of publicly available ischemic ST datasets recapitulated spatial and temporal patterns relevant to glial scar formation observed in our dataset. The lesion core–periphery border was a site of accumulating chemokine expression and *Apoe-Trem2* coexpression, with significant upregulation of reactive glia signatures.

## Discussion

In this study, we mapped the transcriptional landscape during the acute phase of experimental ischemic brain injury, providing a holistic view of the response mediated by a complex network of cellular components (CCs). We showed that anatomical brain regions and lesion areas are robustly captured by ST (Fig. 1). Cell activation, inflammation, and tissue remodeling were hallmarks of the coordinated response to ischemia, with the cellular response organized into layers and reactive glial populations located in the lesion periphery (Fig. 2). We documented the shift in intercellular communication and highlighted the role of *Apoe-Trem2* in microgliosis (Fig. 3). Using single-cell and single-nucleus transcriptomics, we characterized ischemia-associated profiles of glial cells, revealing similarities to reactive states described in neurodegenerative CNS disorders (Figs. 4 and 5). Through reanalysis of published ST datasets, we recapitulated the lesion core–periphery separation and the localization of glial scar-governing gene signatures (Fig. 6).

ST can accurately recover brain anatomy owning to the unique function and cellular composition of each part (24). After ischemic injury, the cortical structure and function are severely disrupted, transforming the cortex into an ischemic core and an enclosing penumbra, which later forms the glial scar (16). The ischemic core is the most affected area, while the penumbra maintains some metabolic and polarization capacity (2, 23, 57). We identified the lesion core as the epicenter of cellular death, accompanied by infiltration of peripheral immune cells and upregulation of genes linked with cell activation, cytokine production, and antimicrobial humoral response (Figs. 1 and 2 and Dataset S2). Early immune cell infiltration was followed by fibroblasts and vasculature cells, restoring microvasculature and initiating fibrotic core formation (1). The resulting inflammation activates astrocytes, microglia, and oligodendrocyte lineage cells, leading to the formation of a dense limiting border known as the glial scar (*SI Appendix*, Fig. S9) (4, 58).

During the 7-d postinjury period, the glial scar matured, evidenced by changes in its thickness, cellular composition, and shifts in communication (Figs. 1–3). On day 1, a gradient of chemokine expression (*Ccl12, Ccl3, Ccl4*) delineated the lesion border from brain parenchyma (Fig. 2 and Dataset S2). Supplemented by deconvolution analysis, we uncovered a reactive microglial population on the lesion border, colocalized with chemokine genes (Fig. 2). Chemotactic microglia produce inflammatory signals after acute injury (35) or near demyelinated lesions of slowly progressing pathology (42), initiating a microglial activation cascade that densifies nearby tissue (36). Another role is to guide locomotion of multiple cell types, including peripheral immune cells (59), neuronal progenitor cells (37, 38), and oligodendrocyte-precursor cells (60). Along chemokines, the shift in intercellular communication toward glia (Fig. 3) and localization of reactive genes (*Serpina3n, Gfap, C4b, Mt1*) indicated early glial activation. The expression of *Serpina3n* is particularly intriguing, considering its inhibitory activity of Granzyme B protease, a potent neuronal toxin expressed by cytotoxic CD8+ T cells (55). Under pathological conditions, *Serpina3n* is expressed by astrocytes (45, 56, 61), oligodendrocytes (44, 62, 63), OPCs (64), and neurons (56, 65), suggesting a defense mechanism against T cell mediated cytotoxicity. In concordance, we detected multiple cellular sources of SERPINA3N (Fig. 5).

On day 3, the lesion area acquired a layered structure composed of different CCs (Figs. 2 and 3). The lesion core underwent intensive remodeling, coordinated by immune cells, vasculature cells, and fibroblasts. The lesion periphery, almost fully enclosing the core, consisted predominantly of microglia and OPCs. One such microglia-guiding signal is the *Apoe-Trem2* interaction (66), leading to metabolic changes, proliferation, and activation-induced hypertrophy, which densify the tissue (Fig. 3 and *SI Appendix*, Fig. S5), isolate the inflamed ischemic core from intact parenchyma, and initiate glial scar formation (16, 36, 48). However, the benefit of microglial activation to ischemia outcome remains debated (26). In a simplified view, activated microglia may adopt either a pro- or anti-inflammatory state (M1/M2) (67), though recent studies suggest a more complex view (42, 43, 45, 61, 62, 68). In our dataset, we identified a small population of reactive microglia (Fig. 4), with genes expressed in early activated microglia of experimental AD models (43). These are considered beneficial early in neurodegeneration but become detrimental as pathology progresses and microglia mature into a chronic reactive state (*Apoe^Hi^, Trem2^Hi^, Cst7^Hi^*). Interestingly, the spatial expression of these genes peaked on day 7, suggesting a shift from beneficial to detrimental phenotype.

The pathological milieu and recruiting microglia affect nearby astrocytes and oligodendrocytes, triggering transcriptional, morphological, and metabolic changes collectively named gliosis (62, 69–71). Both cell populations enveloped the lesion, forming the outer layers of the glial scar (Fig. 2 and *SI Appendix*, Fig. S3), and expressed common reactivity markers (*Gfap, Serpina3n, C4b*; Figs. 4 and 5). The core reactivity signature is shared among glial populations across pathologies including AD (43–45, 61), multiple sclerosis (72, 73), aging (62), and spinal cord injury (30, 64, 74, 75), indicating conserved aspects of activation (41, 70). In accordance, DEGs of reactive populations in our data were enriched for profiles of disease-associated astrocytes and oligodendrocytes (Figs. 4 and 5). A notable observation, evident on day 7, is the expansion of reactive glial signatures into white matter tracts and the striatum (Figs. 4 and 5). This suggests pathophysiological changes outside the cortex, possibly representing early signs of secondary neurodegeneration frequently observed in stroke patients (76).

Oligodendrocytes, once considered passive bystanders, are now documented to have immunomodulatory roles in neurodegeneration (41, 44, 72). In this study, we identified three injury-associated OL populations with temporally and spatially defined roles in ischemic pathology. On day 1, MOL DA2 was the most abundant injury-induced population, accumulated near the lateral ventricle (Fig. 5). Their increased energy demand, translational activity, and expression of genes influencing survival indicate a population priming the OL injury response. With the highest levels of *Serpina3n* and *Klk6*, MOL DA1 population is likely the main source of the anti-inflammatory response. Overexpression of protease inhibitor *Serpina3n* reduces apoptosis and neuroinflammation, stimulating cell proliferation via the Akt-mTOR pathway (56). MOL DA1 also overexpressed *Klk6*, an extracellular protease acting against myelin production (77). Premature myelination can inhibit the regrowth of neurons (78), pointing to a potential regulatory mechanism supporting neuronal reorganization after the injury. Postinjury, MOL IFN gradually strengthened its signature near the lesion core, similar to interferon-responsive populations in microglia (43, 62) and astrocytes (79, 80), indicating active crosstalk to regulate the inflammatory status of the lesion (Fig. 3).

Several limitations should be considered in this work. For practical reasons, our study has a limited design, screening only young male mice during the first week postinjury. Although age- and sex-related risk factors are important for understanding stroke mechanisms and outcome (81), they require analyses in dedicated studies. Analysis of later timepoints is necessary to better understand chronic neuroinflammation, patient recovery, and neurodegenerative disease onset. To mitigate these limitations, we reanalyzed two published ST datasets to increase replicates and extend studied timepoints (Fig. 6). Despite employing different ischemia models, our major biological themes were largely reproduced, helping to elucidate comparability between different models. Despite its limitations, this study provides a complex reference atlas of experimental ischemic brain injury, allowing further data mining and hypothesis testing through reanalysis or interactive exploration at https://scarfweb.nygen.io/eu-central-1/public/xv2x2szz.

We have presented a comprehensive transcriptomic study providing insights into molecular and cellular processes during the acute phase of experimental ischemic brain injury, with temporal, spatial, and single-cell resolution. This work represents a starting point for further studies aiming to elucidate stroke pathology and search for new therapeutic targets.

## Materials and Methods

All animal procedures were performed in accordance with the European Communities Council Directive and approved animal care guidelines. Permanent MCAO were induced in 3-mo-old male mice. MRI, spatial transcriptomics, single-nucleus RNA sequencing, single-cell RNA sequencing, bulk RNA sequencing, and immunohistochemistry were performed at 0, 1, 3, and 7 d postinjury. Detailed reagents, resources, and methodologies essential for reproducing the results are provided in *SI Appendix*.

## Supplementary Material

Appendix 01 (PDF)

Dataset S01 (XLSX)

Dataset S02 (XLSX)

Dataset S03 (XLSX)

Dataset S04 (XLSX)

Dataset S05 (XLSX)

Dataset S06 (XLSX)

## Data Availability

All study data are available and have been deposited in various repositories. The raw sequencing data have been deposited in the NCBI GEO under the accession number GSE233815 (82), the processed sequencing data in Mendeley Data with the identifier 10.17632/gnb2dsjms2.1 (83), the interactive spatial data in Nygen Analytics ScarfWeb at the URL https://scarfweb.nygen.io/eu-central-1/public/xv2x2szz (84), and the code on GitHub at https://github.com/LabGenExp/Spatial_MCAO (85). Additionally, previously published data were used for this work, as referenced in sources (10, 12, 21).
