# Supplementary material for: Spatiotemporal transcriptomic map of glial cell response in a mouse model of acute brain ischemia
Source: Proc Natl Acad Sci U S A. 2024 Nov 5;121(46):e2404203121. doi: 10.1073/pnas.2404203121 (PMC11573666; doi:10.1073/pnas.2404203121)
Supplement: Supplementary file 1 — Appendix 01 (PDF) [file pnas.2404203121.sapp.pdf]

PNAS

Supporting Information for

Spatiotemporal transcriptomic map of glial cell response in mouse model of acute brain ischemia

Daniel Zucha[1,2], Pavel Abaffy[1], Denisa Kirdajova[1,3], Daniel Jirak[4,5], Miroslava Anderova[3], Mikael Kubista[1], and Lukas Valihrach[1,*]

[1] Laboratory of Gene Expression, Institute of Biotechnology of the Czech Academy of Sciences, Vestec, Czech Republic,

[2] Department of Informatics and Chemistry, Faculty of Chemical Technology, University of Chemistry and Technology, Prague, Czech Republic,

[3] Department of Cellular Neurophysiology, Institute of Experimental Medicine of the Czech Academy of Sciences, Prague, Czech Republic,

[4] Department of Radiodiagnostic and Interventional Radiology, Institute of Clinical and Experimental Medicine, Prague, Czech Republic,

[5] Faculty of Health Studies, Technical University of Liberec, Liberec, Czech Republic.

*Corresponding author: Lukas Valihrach

Email: lukas.valihrach@ibt.cas.cz

This PDF file includes:

Materials and Methods

Figures S1 to S9

SI references

Other supporting materials for this manuscript include the following:

Datasets S1 to S6

Materials and Methods

Mice. All procedures involving the use of laboratory animals were performed in accordance with the European Communities Council Directive 24 November 1986 (86/609/EEC) and animal care guidelines approved by the Institute of Experimental Medicine, Academy of Sciences of the Czech Republic (Animal Care Committee on March 15, 2022; approval number 50/2020). All efforts were made to minimize both the suffering and the number of animals used. Experiments were performed on 3-month-old C57Black/6 male mice. In addition, for single-cell RNA-seq and immunohistochemistry we used B6.Cg-Tg(Plp1-cre/ERT)3Pop/J which was cross-bred with B6;129S6-Gt(ROSA)26Sortm14 (CAG-tdTomato)Hze/J (Jackson Laboratory, Bar Harbor) also termed Plp1/tdTomato mouse. The expression of tamoxifen-inducible Cre recombinase is controlled by the Plp1 promoter and after tamoxifen administration, tdTomato red fluorescent protein is expressed in Plp1 positive cells, which includes oligodendrocytes. Tamoxifen was administered intraperitoneally for two days (100 mg/kg, Sigma–Aldrich)(1). The mice were kept on 12-hr light/dark cycles with access to food and water *ad libitum* and assigned randomly to experimental groups.

Middle cerebral artery occlusion (MCAO), a model of experimental stroke. Prior to the induction of MCAO, mice were anaesthetized with 3% isoflurane (Aerrane, Baxter) and maintained in 2% isoflurane using a vaporizer (Tec-3, Cyprane Ltd.). A skin incision between the orbit and the external auditory meatus was made, and a 1-2 mm hole was drilled through the frontal bone 1 mm rostral to the fusion of the zygoma and the squamosal bone, about 3.5 mm ventrally to the dorsal surface of the brain. The middle cerebral artery (MCA) was exposed after the dura was opened and removed. The MCA was occluded by short coagulation with bipolar tweezers (SMT) at a proximal location, followed by transection of the vessel to ensure permanent occlusion. During the surgery, body temperature was maintained at 37 ± 1°C using a heating pad. This MCAO model yields small infarct lesions in the parietal cortical region. Intact cortical tissue from 3 months old mice was used as control. We have evaluated the impact of sham surgery (same surgical procedure, without coagulation of MCA) on gene expression in a previous study Androvic et al.(2), which we did not find differences between naive and sham-operated mice.

Brain dissection. Mice were deeply anesthetized with pentobarbital (PTB) (100 mg/kg, i.p.), and perfused transcardially with cold (4–8°C) isolation buffer containing (in mM): NaCl 136.0, KCl 5.4, HEPES 10.0, glucose 5.5, osmolality 290 ± 3 mOsmol/kg. The forebrain hemisphere was isolated by dissection of the olfactory lobes and the midbrain structure (bregma from ~ +3 to -4 mm).

Magnetic Resonance imaging (MRI). All *in vivo* imaging experiments were collected on a 4.7 T MR scanner (Bruker BioSpec, BioSpin, Ettlingen, Germany) equipped with an optimized ^{1}H radiofrequency surface coil custom-designed and constructed in a magnetic resonance laboratory. Five mice with MCAO-induced lesions were scanned per timepoint (1, 3, and 7 days post injury, respectively), and one additional mouse underwent a repeated scanning at all of the three timepoints. During the scanning, the animals were anesthetized with 1.5% isoflurane mixed with air. The measurement protocol consisted of a standard 2D Rapid Acquisition with a Relaxation Enhancement (RARE) multi-spin echo sequence acquiring T_2-weighted sagittal, coronal and axial images. For volumetry, coronal and axial planes were used with these basic imaging sequence parameters: repetition time (TR) = 3300 ms, effective echo time (TE) = 36 ms, excitation and refocusing pulses = hermite pulses, number of acquisitions (NA) = 8, scan time (ST) = 11 min 52 s, turbo factor = 8, slice thickness = 0.6 mm, field of view (FOV) = 35 mm and spatial resolution = 137 x 137 μm^2. The volumetry of lesioned area and its 3D reconstruction were assessed manually using VGstudio Max (v2.1, Volume Graphics) in each axial and coronal T_2-weighted image (the average from these two planes was calculated). Wilcoxon rank sum test was used for statistical testing of the lesion volume difference. For 3D reconstruction, two stacks of images covering whole mouse brain were acquired with slice thickness = 0.4 mm, ST = 17 m 48s, NA = 12. All other imaging parameters were identical.

10X Visium Spatial transcriptomics.

Tissue preparation and sectioning. Dissected forebrains were embedded in optimal cutting temperature (OCT) medium, rapidly placed on dry ice to freeze, and then transferred to -80°C for a maximum of six weeks of storage. The forebrains were sectioned coronally (10 μm thickness) using Leica CM1950 cryostat (Leica Microsystems). Sections collected for 10X Visium Spatial Gene Expression processing were stored at -80°C.

Section fixation, staining and imaging. Fixation, staining, and imaging was performed strictly according to manufacturer's manuals as in "Methanol fixation + H&E Staining Demonstrated protocol" (CG000160) and "Imaging Guidelines Technical Note" (CG000241). In brief, the sections were shortly incubated to thaw, methanol-fixed, isopropanol-incubated, and H&E stained. Stained sections were imaged using Carl Zeiss AxioZoomV16 upright microscope equipped with PlanNeoFluar Z objective (2,3x magnification, 0,57 NA, 10,6 WD) at total 63,0x zoom. Images were imaged with 5% overlap and stitched using ZEN blue pro 2012 software.

Sequencing library preparation. Sequencing libraries were prepared according to the manufacturer's manual "Visium Spatial Gene Expression Reagent Kits User Guide" (CG000239). In brief, directly after imaging, the sections were permeabilized for 9 minutes, which is the optimal time determined in the "Visium Spatial Gene Expression Reagent Kits - Tissue Optimization User Guide" (CG000238) experiment. When permeabilized, on-slide reverse transcription was performed followed by second strand cDNA synthesis. Next, the double-stranded cDNA was transferred to a microtube, PCR-amplified, enzymatically fragmented, size selected, and tagged with Illumina sequencing adapters. The quality of each library was assessed by capillary electrophoresis on FragmentAnalyzer using the NGS High Sensitivity kit (Agilent, DNF-474). Sample libraries were pooled and sequenced on Illumina NovaSeq 2000 targeting 50,000 read pairs per tissue-covered spot.

Low-level data processing. The raw sequencing data were processed using the recommended set of *Spaceranger* function (v1.2.2, 10X Genomics) for processing of fresh frozen samples. Binary base call files were demultiplexed using *mkfastq* function with default parameters. The resulting fastq files were mapped separately to spaceranger reference (mm10/GRCm38) using *count* function, which takes a microscope slide image and fastq files, performs alignment, tissue detection, fiducial detection, and barcode/UMI counting.

Data processing and clustering – control section. The analysis was performed using R-based *Seurat* pipeline (v4.1.0, https://github.com/satijalab/seurat)(3), accessible on https://github.com/LabGenExp/Spatial_MCAO). First, we counted quality metrics, excluded spots with less than 100 unique genes from the analysis and normalized the counts with the *SCTransform* function using the maximal number of identified variable features. We performed principal component analysis (PCA) for the top 100 principal components (PCs) on the genes of nuclear origin. Determined the optimal number of PCs by inspecting their standard deviations (*ElbowPlot* analysis) and spot PC scores (*DimHeatmap* analysis) to determine the variance they capture. We used the top 25 PCs as input for the UMAP dimensionality reduction. Next, we determined the clusters using functions *FindNeighbors* and *FindClusters* with the Leiden algorithm for modularity optimization. We examined the clusters at varying resolutions (from 0.6 to 2.0), visually identifying the robustness in the UMAP and Spatial plots. We used a resolution of 1.2, yielding 19 clusters, as baseline, which produced similar annotations to the Allen Brain Atlas (https://mouse.brain-map.org/static/atlas, images 64 and 65, Reference Atlas version 2, 2011)(4). Additionally, the anatomically related clusters were merged, yielding a final 13 distinct brain region-specific clusters.

Region annotation and comparison to Allen Brain Atlas. We calculated differentially expressed genes (DEGs) for the individual brain regions (i.e., region vs. rest of the section) using the *FindAllMarkers* function. The DEG was considered as a brain region marker, when passing the criteria of $\log_2$-fold change > 0.58 and adjusted p-value < 0.01. Top 100 regional Allen Brain Atlas

(ABA) *in-situ hybridization* markers (fold-change sorted) were obtained for each region separately (https://mouse.brain-map.org/search/index). The significances of overlaps between our top 100 log_2-fold change sorted and top 100 ABA markers were calculated using hypergeometric test (*phyper* function, *stats* R package, v 4.2.2) with the full gene list of corrected count matrix as background set (n = 18302 genes). P-values were corrected for multiple hypotheses testing by the Benjamini-Hochberg procedure (false-discover rate, FDR).

Data processing and clustering – all sections. Spatial data were collected in two batches: Batch 1 consisted of a control (bregma -1.3 mm) and post-ischemic day 7 sections (bregma +0.5 mm); Batch 2 were three post-ischemic sections collected on day 1, day 3 and day 7 (all bregma -1.3 mm). We filtered the low-quality spots (> 200 unique genes), normalized the datasets separately by batches (*SCTransform*), identified the maximal number of integration features (*SelectIntegrationFeatures*), computed the missing sctransform residuals (*PrepSCTIntegration*), identified the integration anchors (*FindIntegrationAnchors*), and merged the two datasets (*IntegrateData*). Using the matrix of centered, corrected Pearson residuals we calculated the top 100 PCs using the list of genes of nuclear origin. Using *ElbowPlot* and *DimHeatmap* analyses, we chose the top 30 PCs as being most informative and used them as input for the UMAP dimensionality reduction. Clusters were determined using the *FindNeighbors* and *FindClusters* functions. We examined clustering robustness at varying resolution parameter values (0.6 to 2.0) and settled for the resolution of 1 yielding 27 clusters, which we annotated into 16 anatomically related brain regions. Next, we repeated the integration process as outlined above for the spots originating from the regions of the cortex and from the ischemic lesion. After data merger, top 20 PCs were used in the UMAP clustering, yielding 19 clusters. Out of these six defined injury-induced clusters. Finally, we combined clustering from the whole-section and cortical integrations, resulting in the final annotation of 16 brain regions and 6 ischemic areas. For the final presentation, we excluded the post-ischemic day 7 section (bregma +0.5 mm), due to its different localization. The section was, however, included in the integration, to allow for mutual anchoring of lesion clusters.

Pathway enrichment. Using the *FindAllMarkers* function we calculated DEGs for each annotated region (log_2 fold-change threshold > 0.58 and adjusted p-value < 0.01). On the list of marker genes, we performed the Gene Ontology (GO) analysis of biological processes, cellular components, and molecular functions, using the *enrichGO* function (*clusterProfiler* package v4.2.2), with the 18302 genes as background and FDR-adjustment of p-values (FDR < 0.01). For pathway enrichment we used Metascape(5) individually for each section to generate the networks of commonly enriched terms in the ischemic regions. We recapitulated the enrichment pathway settings, searching for GO biological processes with minimal overlap of five genes at a significance level of <0.01. The network maps were generated automatically with the Metascape tool, for which we reviewed the cluster annotation based on the GO terms included within the parent cluster.

Re-analysis of the Zeng et al. single-cell dataset. We obtained the Zeng et al. (6) dataset from GEO omnibus (GSE227651) and re-analyzed it using R-based *Seurat* pipeline (v4.1.0, https://github.com/satijalab/seurat)(3). We filtered the set for cells with >200 unique transcripts and mitochondrial RNA < 15%, log-normalized and scaled the data *(NormalizeData* and ScaleData functions, respectively*)*. We computed PCA using 50 PCs, then using the *ElbowPlot* and *DimHeatmap* analyses, we identified the top 25 PCs to account for sufficient variation in the data. The dataset was then integrated across the samples using Harmony integration (7). Clusters were calculated using *FindNeighbors* and *FindClusters* functions with the Louvain's algorithm for modularity optimization at a resolution of 1. The procedure yielded 36 clusters, which we annotated to 15 different cell types considering their marker expression (*FindAllMarkers,* $log_2FC > 0.58$, padj < 0.01).

RCTD deconvolution. We performed the Robust Cell Type decomposition (RCTD) deconvolution using the *spacexr* R package v2.0.0. We prepared the *Reference object* from the raw count matrix of the in-house re-analyzed Zeng et al. (6) dataset. For the spatial data, we prepared a separate *SpatialRNA object* from each section separately. *SpatialRNA objects* were similarly prepared from

the raw RNA count matrix (‘*Spatial’* assay in the *SeuratObject*) and their spot coordinates. Next, we created a separate *RCTD object* for every section using *create.RCTD* with the *Reference object* and *SpatialRNA object* as inputs. We deconvoluted the *RCTD objects* using *run.RCTD* with the doublet mode parameter set to ‘multi’ to allow fitting the number of cell types per spot. This resulted in a matrix of ratios of cell type levels per spot. To obtain cell type-gene correlation matrix, the per-spot cell type ratios were correlated with gene expression using *cor.matrix(method = “pearson”)* separately for each timepoint.

Signature expression. To visualize a module of common genes, either as a set of population markers or a biological process, we used *AddModuleScore* function (*Seurat* package), which compares the average expression of the signature genes with permuted randomly selected genes of similar expression levels (size = 100 genes, 24 permutations). To visualize populations described in this study, we used top 15 markers (>0.58 log2FC sorted, P_{adj} < 0.01) for the populations described in this study, and full marker gene lists for the published populations. To visualize GO terms, we used all its DEGs that passed the marker criteria (log_2-fold change > 0.58 and adjusted p-value < 0.01).

Spatially-resolved ligand-receptor analysis with SpaTalk. We predicted ligand-receptor cell-cell communications using the *SpaTalk* R package v1.0. When inspecting either full cortex in control section or lesion periphery in the lesions, we prepared a separate *SpaTalk* object for each section (*createSpaTalk*) by providing un-normalized count matrix and metadata on spot location and cell content calculated with RCTD. Having provided reference single-cell cell type profiles by re-analyzed Zeng et al. dataset, we decomposed the spatial spots into single cells (*dec_celltype*), identified all putative cell-cell interactions (*dec_cci_all*) and identified downstream affected genes and pathways (*find_lr_path*), all performed with functions at default arguments. To visualize the change in the flow of intercellular communication, we visualized the total number of mutual interactions using *circlize* R package v0.4.16. The ligand-receptor co-expression was visualized as the smaller expression value from the gene pair, under the condition that both had expression different from zero. Genes identified to be downstream affected by Apoe-Trem2 signaling were subjected to gene ontology enrichment by *enrichGO* function from the *clusterProfiler* package and identified as enriched at p-value cutoff 0.01 after FDR-adjustment.

Reanalysis of Spatial Transcriptomics datasets from Han et al. 2024 and Scott et al. 2024. We analyzed datasets from Han et al. 2024 (8) and Scott et al. 2024 (9) analogically to our spatial dataset. In short, the datasets were downloaded from their respective public repositories, made into seurat objects, quality control checked, filtered for low quality spots (removing nFeature or nCount < 100), log-normalized (*NormalizeData*), dimensionlity reduced by to top 20 PC components, clustered at resolution = 1.2, brain regions annotated using markers from Allen Brain Atlas and our dataset, and RCTD deconvoluted with the same Zeng et al. reference. The chemokine and reactive expression modules were computed identically to ours, using the *AddModuleScore* function for the three CC chemokine genes (*Ccl12, Ccl3, Ccl4*) or top 15 DEGs of that population. Apoe-Trem2 co-expression was also visualized identically, showing the lower expression of the ligand-receptor pair.

Interactive spatial transcriptomics data exploration. Our spatial transcriptomics data including were made accessible for interactive exploration at https://scarfweb.nygen.io/eu-central-1/public/xv2x2szz. This allows to inspect many metrics in both the UMAP and spatial layouts, including brain regions DEGs, per-spot quality control metrics, cell type proportions, module scores for enriched parent Gene Ontology terms, co-expression values for top 10 numerous ligand-receptor pairs, module scores of Apoe-Trem2 downstream processes, and module scores for reactive glia signatures.

Bulk RNA-Seq

RNA isolation, library preparation and sequencing. Samples were collected from the injured cortical region (including affected as well as non-affected brain tissue) and homogenized using the TissueLyser (QIAGEN). Total RNA was extracted with TRI Reagent (Sigma-Aldrich) according to the manufacturer's protocol and treated with TURBO DNA-free kit (Thermo Fisher). RNA quantity and purity was assessed using the NanoDrop 2000 spectrophotometer (Thermo Fisher) and RNA integrity was assessed using the Fragment Analyzer (Agilent). All samples had RQN > 8. Libraries were prepared from 400 ng total RNA with QuantSeq 30 Library Prep Kit FWD (Lexogen) according to manufacturer's protocol. 1 µl of ERCC spike-in (c = 0.01×; Thermo Fisher) per library was included. Libraries were quantified on the Qubit 2 fluorometer (Thermo Fisher) and Fragment Analyzer (Agilent), and sequenced on the NextSeq 500 high-output (Illumina) with 85 bp single-end reads. 11.5 – 38 million reads were obtained per library with a median of 16 million reads.

Data processing, mapping, and counting. Adaptor sequences and low-quality reads were removed using TrimmomaticSE v0.36(10). Reads mapping to mtDNA and rRNA were filtered out using SortMeRNA v2.1 with default parameters(11). The remaining reads were aligned to GRCm38 and ERCC reference using STAR v2.5.2b with default parameters(12). Mapped reads were counted over Gencode vM8 gene annotation using htseq-count with union mode for handling of overlapping reads(13).

Differential expression and pathway enrichment. We performed differential expression testing using DESeq2 v1.34.0(14). We filtered the data for genes with >10 reads and normalized them by *varianceStabilizingTransformation* function. We applied the DESeq model using *DESeq* function with type of fit parameter set to 'local'. Statistical testing was done by comparing the control sample with each timepoint individually. Genes with $\log_2$ fold-change >0.58 and adjusted p-value <0.01 were considered as differentially expressed markers.

Apoe-Trem2 downstream processes visualized. Genes significantly affected by Apoe-Trem2, representative of highlighted pathways and related to microgliosis, were used to compute respective pathway module scores. Inspired by *Seurat*'s *AddModuleScore* function, the difference in the mean expression between the pathway's and randomly selected gene sets was calculated and min-max normalized per the sample set. The statistical difference between the uninjured and MCAO samples were then determined by Wilcoxon's rank sum test with Benjamini-Hochberg correction.

Bulk deconvolution and validation of deconvolution. Bulk data were deconvoluted using CibersortX algorithm (15) at cibersortx.stanford.edu. The dataset of Zeng et al. (6) served as a reference to build the reference expression matrix of minimum of 160 cells for a cell type. The signature matrix was computed in default mode (quantile normalization disabled, minimal expression of 0.75, replicates of 5, sampling of 0.5). Cell type proportions were computed at default settings with S-mode batch correction enabled, quantile normalization disabled and number of permutations set to n = 500. The statistical difference in abundace of neurons or glia populations in respect to injury and time was modeled as following: *lm(cell type proportion ~ time * condition)* using R's *stats* package v4.3.2. To assess the significance of similarity for cell type proportions in spatial and bulk data, we calculated the concordance correlation coefficient (ccc) as a measure of y = x goodness of fit. For spatial data, for each section we calculated mean cell type proportions across its spots. For bulk, we calculated mean cell type proportions across condition's samples. The ccc was then calculated using *epi.ccc* function from *epiR* package v2.0.74.

Single-nucleus RNA-Seq

Sample and sequencing library preparation. Injured forebrain hemispheres were frozen on dry ice and stored at -80 °C until processing. For each condition, three hemispheres were collected and pooled during the single-nucleus suspension preparation. All steps for nuclei isolation were performed on ice, with instruments and plastics pre-chilled on ice, or centrifugation steps at 4°C. The frozen brain tissues were combined with 2 ml of lysis buffer (10 mM Tris, pH = 7.4; 10 mM

NaCl; 3 mM $MgCl_2$; 0.1 % NP-40; 0.2 U/μl RNaseOUT; 0.32 M Sucrose; 1x protease inhibitor, Roche) and transferred to 2 ml Dounce tissue grinder (Sigma). Tissues were homogenized by 20 strokes with pestle A, followed by 20 strokes with pestle B and filtered using 30 μm filter (Celltricks). Tissue grinder was rinsed with 1 ml HEB buffer (Hibernate A, ThermoFisher; 10 μl/ml GlutaMAX, ThermoFisher; 0.32 M Sucrose) and the wash filtered into the same tube. Homogenates were split by careful pipetting of 1.2 ml into two 2 ml tubes prefilled with 500 μl sucrose cushion (1.2 M sucrose; 10 mM Tris; 10 mM NaCl; 3 mM $MgCl_2$; 0.2 U/μl RNase OUT) and centrifuged at 13000 g for 20 minutes at 4°C. Supernatants were removed, except 100 μl to keep the pellets covered. Pellets with the remaining supernatant were resuspended in 1 ml sucrose solution (1.0 M sucrose; 10 mM Tris; 10 mM NaCl; 3 mM $MgCl_2$; 0.2 U/μl RNase OUT), pipetted into 2 ml tubes prefilled with 500 μl sucrose cushion and again centrifuged at 13000 g for 20 minutes at 4°C. Supernatants were removed and the pellets resuspended in 1 ml of Nuclei Wash Buffer (DPBS without calcium and magnesium; 0.5 % BSA; 0.2 U/μl RNase OUT; all ThermoFisher) and transferred into 1.5 ml tubes. Samples were centrifuged at 500 g for 5 minutes, the pellets resuspended in 1 ml of Nuclei Wash Buffer and centrifuged again. The final pellets with nuclei were resuspended in 1 ml of Nuclei Wash Buffer and filtered using 30 μm filter. Nuclei stained with Propidium Iodide were counted using CellDrop Automated Cell Counter (Denovix) at default settings. Nuclei suspensions were diluted to final concentrations of 1000 nuclei/μl and single-nucleus RNA-Seq (snRNA-Seq) libraries were prepared using Chromium Next GEM Single Cell 3' Kit v3.1 (1000268, 10X Genomics) according to the manufacturer's protocol. Final libraries were eluted with EB buffer with 0.1% Tween, and their quality was assessed by capillary electrophoresis on the FragmentAnalyzer using the NGS High Sensitivity kit (DNF-474, Agilent). The libraries were pooled and sequenced on an Illumina NovaSeq 2000. Approximately 100M reads per sample were obtained.

Data processing. Libraries were controlled for contamination using fastq_screen v0.11.1. Reads were mapped against the mouse genome GRCm38 and counted using annotation genecode.vM8 using STAR v2.7.3a with parameters "--soloType CB_UMI_Simple --soloFeatures GeneFull –soloCBmatchWLtype 1MM_multi_pseudocounts –soloUMIdedup 1MM_Directional –soloUMIfiltering MultiGeneUMI". Empty droplets were filtered out using *emptyDrops* command from *DropletUtils* package (v 1.14.1)(16) with lower parameter set to 1000. All droplets with FDR < 0.001 were considered as nuclei. UMI count matrix was processed using the *Seurat* pipeline (*Seurat* v4.1.0, https://github.com/satijalab/seurat)(3), accessible here: LabGenExp Github). Background RNA was removed using SoupX package (v 1.5.2)(17) set at the default parameters. UMI counts were normalized using *SCTransform* and *Seurat* integrated. Nuclei were clustered using top 30 PCs and visualized using UMAP. In total, 26 clusters were identified and manually annotated based on the expression of known markers. Next, neuronal and non-neuronal cells were separated and processed for detailed annotation. Cells across the samples were merged, *SCTransform*-ed, clustered and re-annotated into the major populations.

Data analysis. Three populations were selected from non-neuronal populations, astrocytes (including ependymal cells and neuroblastema cells), microglia, and oligodendrocyte-lineage cells, and analyzed them separately using the *Seurat* pipeline (LabGenExp Github). UMI counts were normalized, *SCTransformed*, clustered and annotated. DEGs were calculated (*FindAllMarkers* function) and population markers defined (DEGs with $\log_2$ fold-change threshold > 0.58 and adjusted p-value < 0.01). On the list of marker genes, we performed the Gene Ontology (GO) analysis of biological processes, cellular components, and molecular functions, using the *enrichGO* function (*clusterProfiler* package v4.2.2), with the size of full gene list of the normalized matrix as for the background set, and selected the significant process based on the FDR-adjusted p-values (FDR < 0.01). Signature expression of the identified populations were visualized in the spatial sections as described in the *Spatial transcriptomics – Signature expression* Method's section.

Metadata analysis. Markers of the published populations were obtained from the respective materials provided by the study's authors. Signature expressions were calculated using *AddModuleScore* function (*Seurat* package), which compares the average expression of the

signature genes with a permuted randomly selected genes of similar expression levels (size = 100 genes, 24 permutations). The entire signature gene list was used to calculate the signature. To calculate marker overlaps, the obtained list of markers was filtered for genes present in the normalized SCT matrix, and then significance tested using the hypergeometric test (*phyper* function), with the full gene list as background set.

Single-cell transcriptomics

Sample preparation. The Plp1/tdTomato mice were deeply anesthetized with pentobarbital (PTB) (100 mg/kg, i.p.), and perfused transcardially with a cold (4–8°C) isolation buffer containing (in mM): NaCl 136.0, KCl 5.4, HEPES 10.0, glucose 5.5, osmolality 290±3 mOsmol/kg. To isolate the control and ischemic areas, the brain was sliced into 600 µm coronal sections using a vibrating microtome Leica VT1200S (Leica Microsystems). The collected tissue was incubated with continuous shaking at 37°C for 45 min in 1 ml of papain solution (20 U/ml) and 0.2 ml DNase (both from Worthington) prepared in isolation buffer. After papain treatment, the tissue was mechanically dissociated by gentle trituration using a 1 ml pipette. The dissociated cells were layered on top of 5 ml of ovomucoid inhibitor solution (Worthington) and harvested by centrifugation (70 x g for 6 min). This method routinely yielded ~2 x 10^6 cells per mouse brain. Cell aggregates were removed by filtering with 70 µm cell strainers (Becton Dickinson). Three animals per condition were pooled in the preparation of cell suspension. After obtaining an aliquot for FACS, the cell suspension was spin down, concentrated, and used for library preparation.

Collection of single cells. Single cell suspension from Plp1/tdTomato mice were sorted using fluorescent activated cell sorting (FACS; BD Influx). The flow cytometer was manually calibrated to deposit a single cell in the center of collection tube. Hoechst 33258 (Life Technologies) was added to the suspension of cells to check viability. 3000 Plp1+ cells from each group (control, D1, D3, D7) were collected into 96-well plates (Life Technologies) coated with BSA (ThermoFisher Scientific) and containing 5 µl of DMEM (ThermoFisher Scientific) and 15% FBS (HyClone).

Library preparation, data processing and analysis. Single-cell RNA-Seq (scRNA-Seq) libraries were prepared using Chromium Next GEM Single Cell 3' Kit v3.1 (1000268, 10X Genomics) according to the manufacturer’s protocol. Final libraries were eluted with EB buffer with 0.1% Tween, and their quality was assessed by capillary electrophoresis on a FragmentAnalyzer (Agilent) using the NGS High Sensitivity kit (DNF-474). The libraries were pooled and sequenced on an Illumina NovaSeq 2000. Low-level data processing was performed similarly as for the snRNA-Seq data, with an exception in a STAR parameter “--soloFeatures Gene”. UMI count matrix was processed using *Seurat* pipeline (*Seurat* v4.1.0, https://github.com/satijalab/seurat)(3). In brief, UMI counts were normalized (*SCTransform*), PCA-reduced, UMAP visualized, clustered (*FindNeighbors, FindClusters*), and annotated based on canonical markers. The dataset was then *Seurat* integrated with the single-nucleus counterpart. The analysis was further performed on the SCT-normalized corrected matrix, using identical set of tools as in the single-nucleus data Method’s section. For further details refer to https://github.com/LabGenExp/Spatial_MCAO.

Immunohistochemistry

Sample preparation and imaging. For immunohistochemical analyses the animals were deeply anesthetized with PTB (100 mg/kg, i.p.) and perfused transcardially with 20 ml of saline followed by 20 ml of cooled 4% paraformaldehyde (PFA) in 0.1 M phosphate buffer. The brains were dissected out, post-fixed overnight with PFA, and treated with a sucrose gradient (ranging from 10% to 30%) for cryoprotection. Coronal 30-µm-thick slices were prepared using a cryostat (Leica CM1950, Leica Microsystems). For immunohistochemical staining, the slices were washed in phosphate-buffered saline, followed by blocking of the non-specific binding sites with 5% Chemiblocker (Millipore) and 0.2% Triton in phosphate-buffered saline. The blocking solution was also used as the diluent for the antisera. The slices were incubated with the primary antibodies overnight, and the secondary antibodies were applied for 2 hours at 4-8°C.The following primary

antibodies were used: mouse anti-GFAP, 1:300; coupled to Alexa 488 (Ebioscience), rabbit anti-Aldh1l1, 1:500 (Abcam), rabbit anti-CathepsinB, 1:500 (Cell Signaling), goat anti-Iba1, 1:500 (Abcam), goat anti-Pdgfrα, 1:500 (RnDsystem), rabbit anti-Col1a, 1:500 (Abcam), goat anti-Serpina3n, 1:100 (Biotechne), rabbit anti-Tnfrsf1a, 1:500 (RnDsystem), and rabbit anti-B2m, 1:500 (Abcam). The secondary antibodies were goat anti-rabbit IgG or goat anti-mouse IgG conjugated with Alexa Fluor 488, 594, or 647 (ThermoFisher). All chemicals were purchased from Sigma-Aldrich unless otherwise stated. An Andor Dragonfly 503 spinning disk confocal microscope with a 20x oil objective was used for the immunohistochemical analysis. Tile scans (15x15) of one hemisphere with stacks of consecutive confocal images were taken at intervals of 2 µm. Stitching of the tile scans was performed automatically by acquisition software Fusion (Oxford Instruments) with 10% overlap. Maximum z-projection images were made using Imaris (Oxford Instruments).

Cell Counting (PLP1 and SERPINA3N, TNFRSF1A, B2M). To determine the number of cells, confocal images (15000 µm x 15000 µm x 30 µm) were taken covering the whole hemisphere from the brain coronal slices. These were prepared from the Plp1/tdTomato control mice and from the mice 1, 3, and 7 days after ischemia (3 animals from each group, 3 brain slices) and stained for the primary antibodies mentioned above. The percentage of double-positive cells for Plp1/tdTomato and SERPINA3N, TNFRSF1A, and B2M were counted in the region of ischemia (core and glial scar) and the corresponding area in the control mice (Dataset S6). Statistical significance between control and injured samples was assessed using unpaired two-sided Wilcoxon rank sum test.

Colocalization (GFAP and ALDH1L1, IBA1 and CTSb). To obtain the co-distribution of stained biological components, confocal images (15000 µm x 15000 µm x 30 µm) were taken covering the whole hemisphere from the brain coronal slices. These were prepared from the WT control mice and the mice 1, 3, and 7 days after ischemia (3 animals from each group, 2 brain slices) and double-stained for the combination of primary antibodies mentioned above. To quantify the overlap of double stains in 3D, a powerful module – ImarisColoc (Oxford Instruments) was used. The percentage of colocalization was calculated from a new channel that contained only voxels that represent the colocalization result in the ischemic and control brain slices (Dataset S6). Statistical significance between control and injured samples was assessed using unpaired two-sided Wilcoxon rank sum test.

Data and code availability

- Raw sequencing data are available at NCBI GEO under accession number GSE233815 (https://www.ncbi.nlm.nih.gov/geo/query/acc.cgi?acc=GSE233815).
- Processed data files are available at Mendeley Data under doi:10.17632/gnb2dsjms2.1 (https://data.mendeley.com/preview/gnb2dsjms2?a=1e744314-eb08-4c66-abe5-e3885b8415c7).
- The code is available on GitHub under the LabGenExp repository Spatial_MCAO (https://github.com/LabGenExp/Spatial_MCAO).
- The spatial data are available for interactive browsing at Nygen Analytics ScarfWeb at https://scarfweb.nygen.io/eu-central-1/public/xv2x2szz.

SI Figures

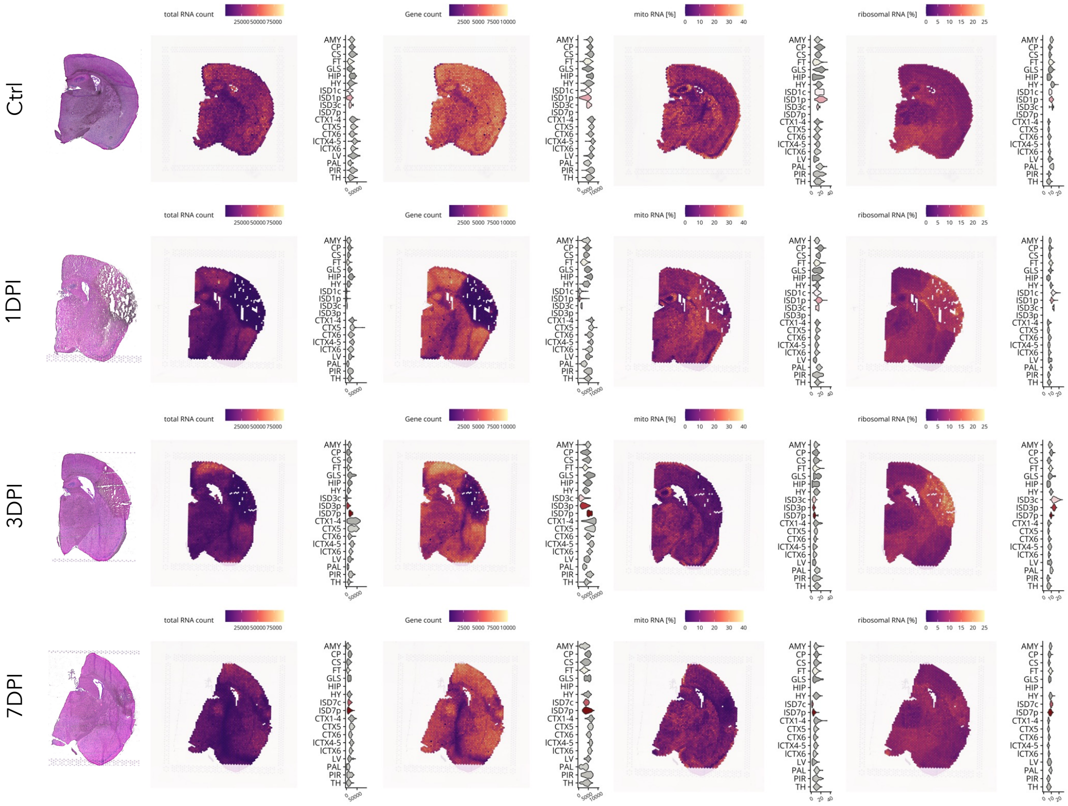

Fig. S1. Quality control metrics of spatial transcriptomics dataset. Hematoxylin-eosin (H&E) stained images of post-MCAO coronal mouse brain sections in the respective timepoints. The sections were collected from a location of bregma ~ -1.3 mm ± 0.1 mm. The spatial plots show basic quality control metrics, including total unique molecular identifiers (UMI) count (total RNA count), gene count, and percentage of mitochondrial RNA (mito RNA, in %) and percentage of ribosomal RNA (in %). In the right-side violin plots, distributions of the metric values in the individual annotated brain regions are shown.

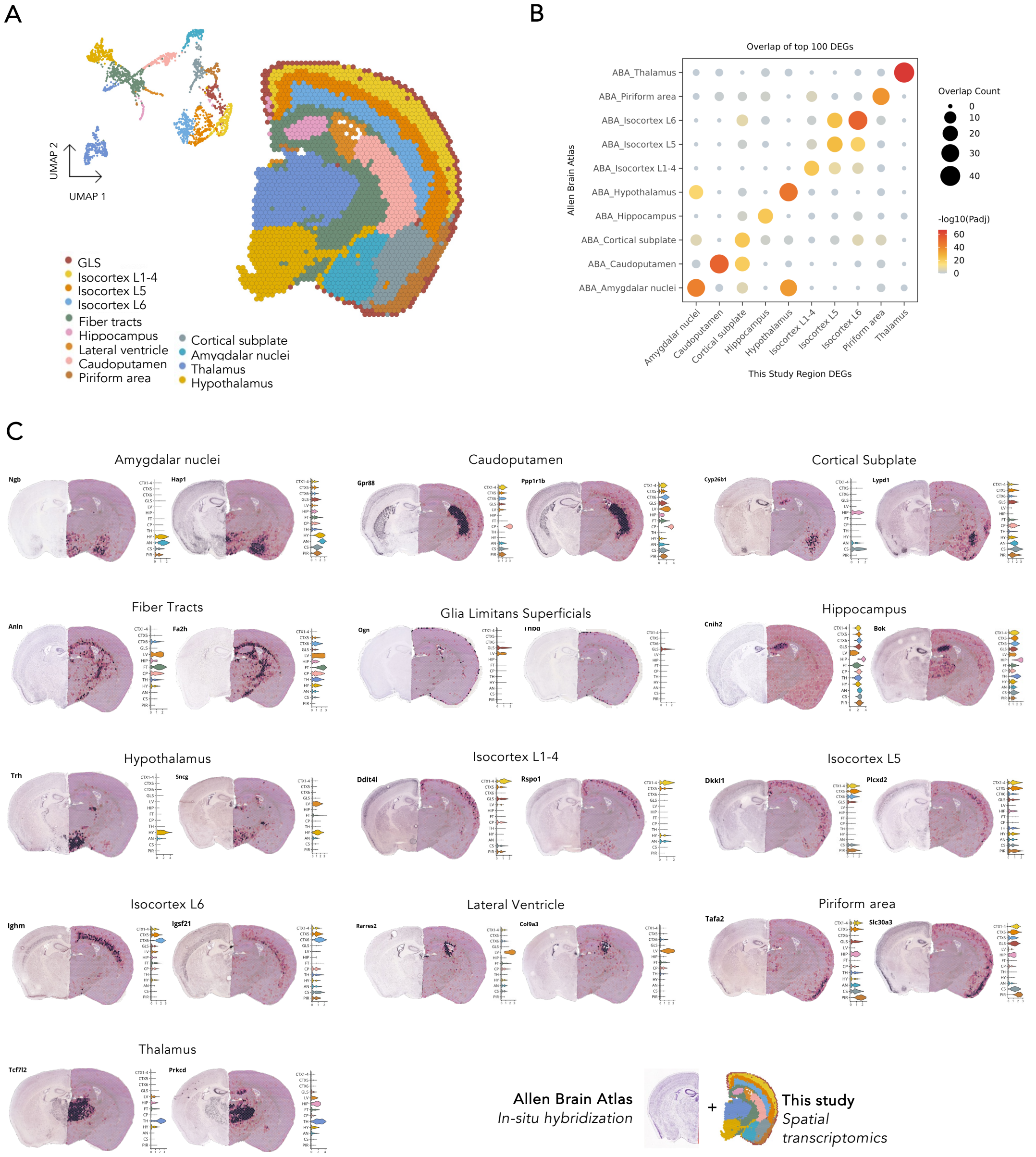

Fig. S2. Region-specific expression of mouse brain markers. A) Left inset: UMAP of the spatial spots of the control section, color-coded based on brain region. Right: spatial plot color-coded based on the brain regions. The color scheme is shared between the plots. **B**) Overlap between top 100 brain region markers as identified by differential gene expression analysis and in the ISH Allen Brain Atlas (Dataset S1). The size (circle size) and the significance (color scale, FDR-adjusted p-value) are plotted. **C**) Combined images of expression for selected markers, combining Allen Brain Atlas *in-situ* hybridization atlas (left, https://mouse.brain-map.org/static/atlas, images 64 and 65, Reference Atlas version 2, 2011, Sunkin et al. (4)) and this study controls section (normalized expression). Distributions of the marker's normalized expression per brain region is shown in the right-side violin plots. The image locations correspond to bregma ~ -1.3 mm.

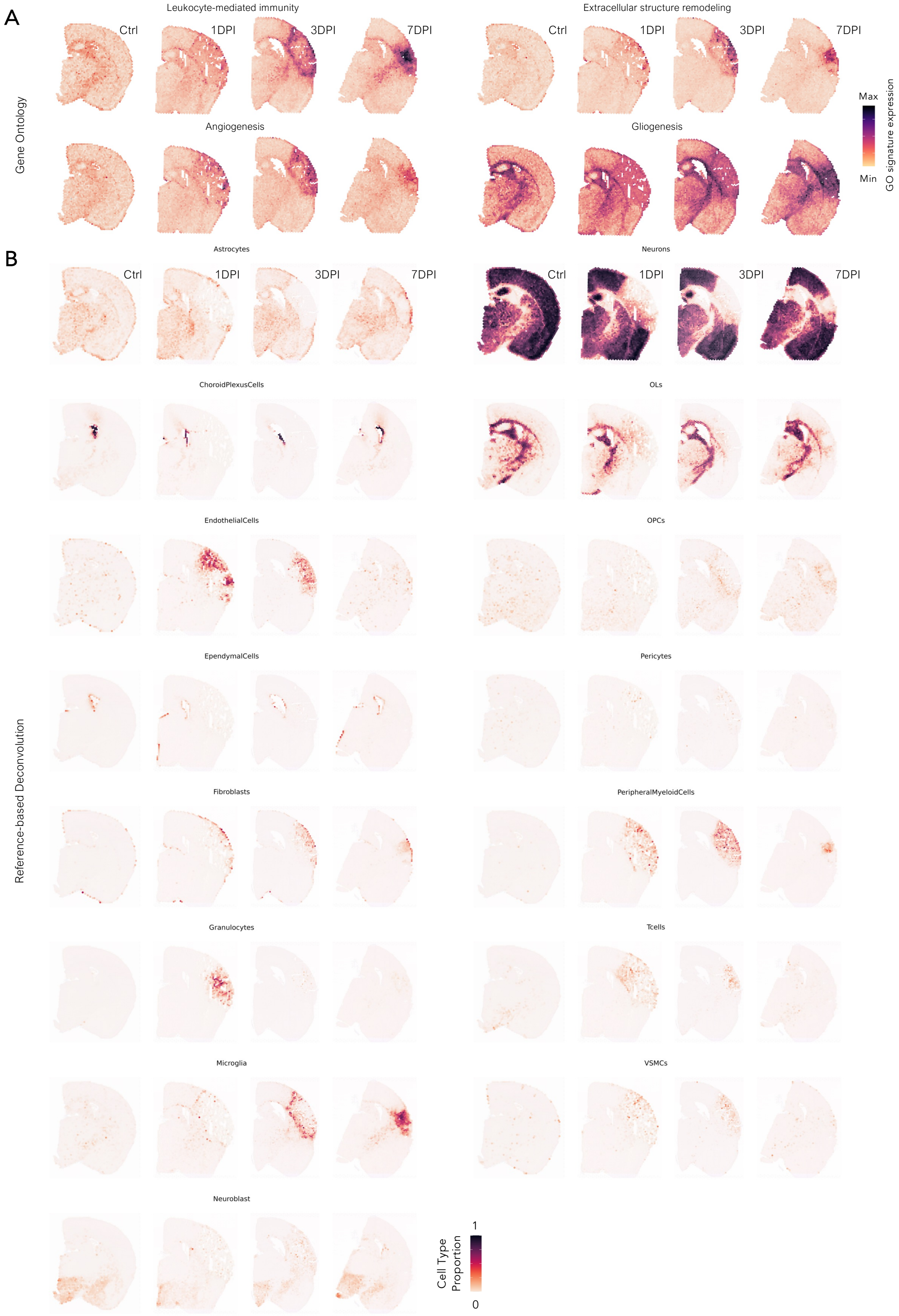
A
Leukocyte-mediated immunity
Extracellular structure remodeling
Ctrl
1DPI
3DPI
7DPI
Angiogenesis
Gliogenesis
Gene Ontology
Max
Min
GO signature expression
B
Astrocytes
Neurons
ChoroidPlexusCells
OLs
EndothelialCells
OPCs
EpendymalCells
Pericytes
Fibroblasts
PeripheralMyeloidCells
Granulocytes
Tcells
Microglia
VSMCs
Neuroblast
Reference-based Deconvolution
Cell Type Proportion
1
0

Fig. S3. (previous page) Spatial localization of selected processes and cell types. A) Projection of module scores calculated by comparing the expression of lesion DEGs belonging to selected enriched terms against randomly sampled genes. **B**) Projection of predicted cell type proportions using reference-based deconvolution algorithm RCTD (18) with single-cell reference from Zeng et al. (6).

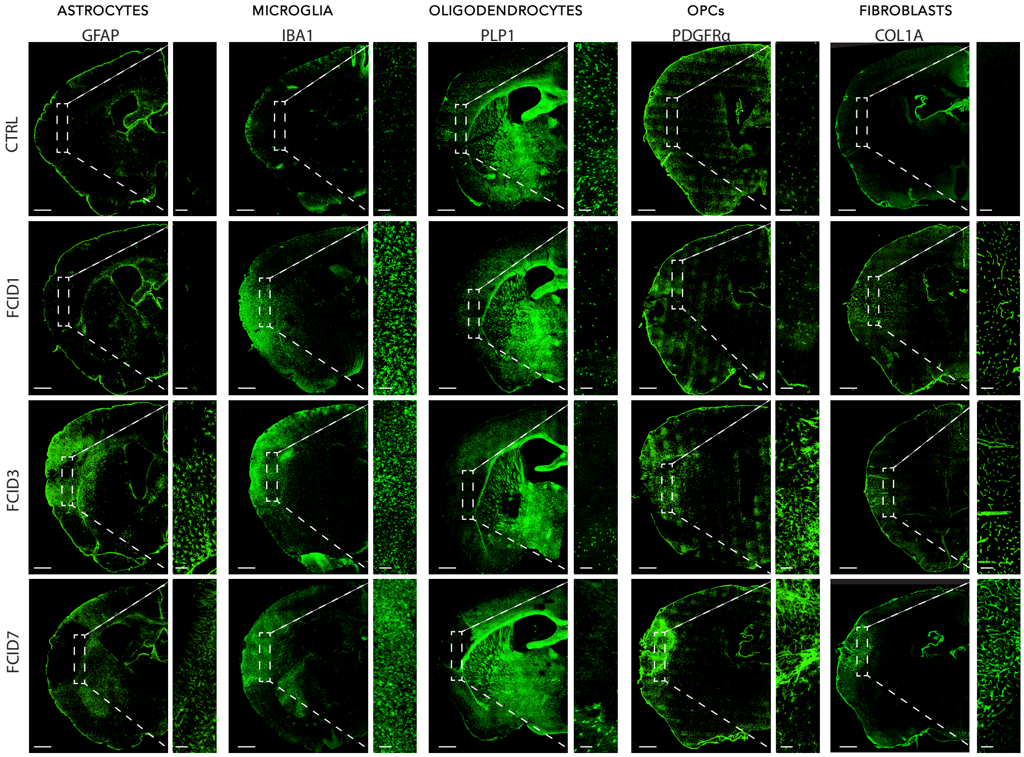

Fig. S4. Validation of cell population presence using immunohistochemistry. Coronal sections of the ipsilesional hemisphere following MCAO injury in mice, antibody stained with representative cell type markers. Scale bars: 500 μm for the hemisphere image, 100 μm for the inset image.

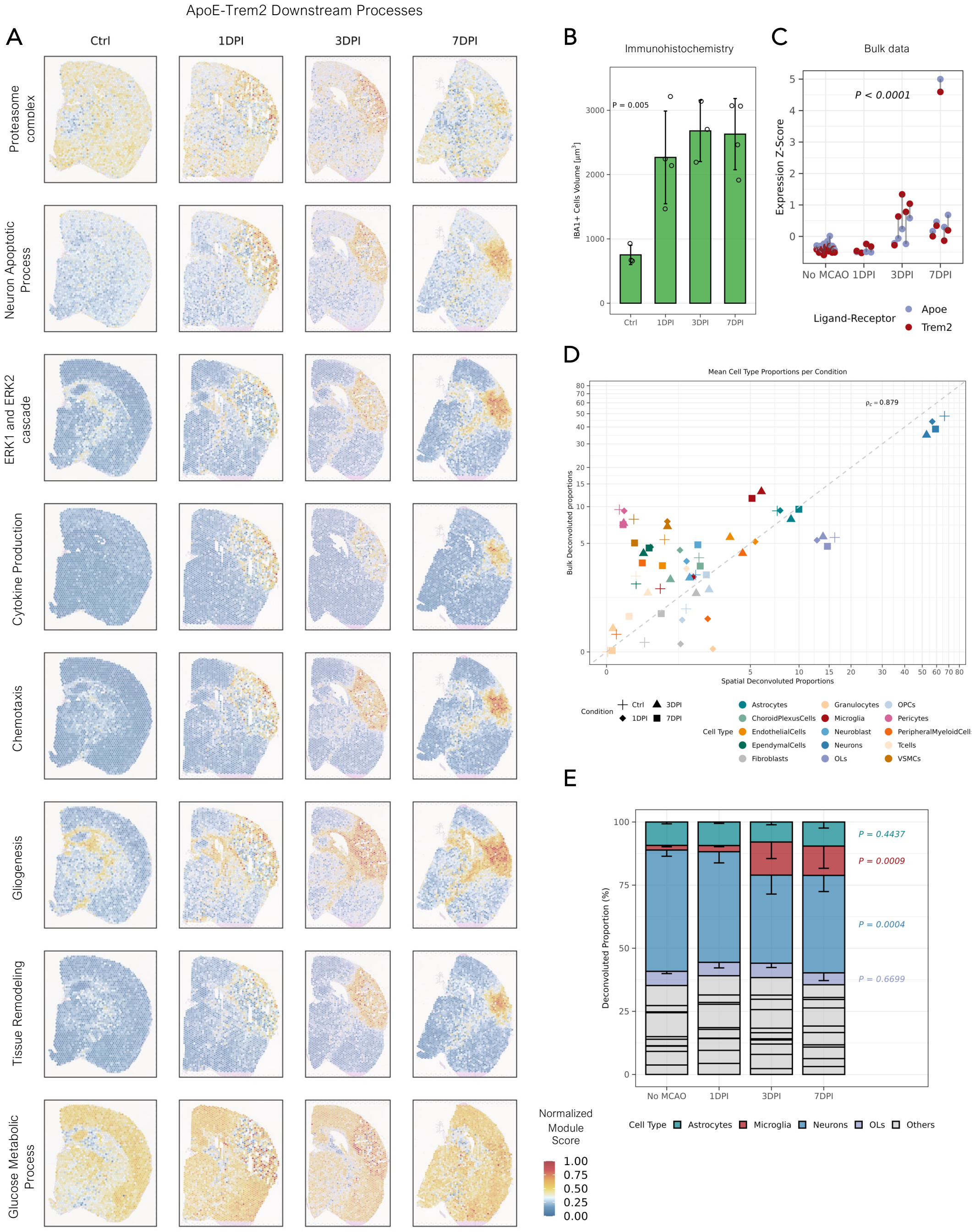

Fig. S5. ***Apoe-Trem2* downstream processes and validation of spatial data. A**) Module scores for enriched processes predicted to be downstream activated after *Apoe-Trem2* interaction. Module scores reflect the difference in average expression of the process and randomly selected genes. Module scores were min-max normalized for each process separately. **B**) Volume IBA1+ cells (microglia) in respective conditions (minimum n = 3 mice per condition, each in minimum n = 2 sections). The significance of volume difference between control and injured mice was determined by Wilcoxon rank sum test. Error bars show standard deviation. **C**) Normalized expression of *Apoe* and *Trem2* in bulk data. **D**) Comparison of cell type proportions as average over spatial section (x

axis) and bulk samples (y axis). Concordance correlation coefficient was calculated to evaluate the closeness to x = y goodness of fit. **E**) Relative cell type proportions in bulk data per condition (with standard deviations shown) with neurons and glia highlighted. Linear model celltype ~ time * condition was used to assess the significance of change in proportion.

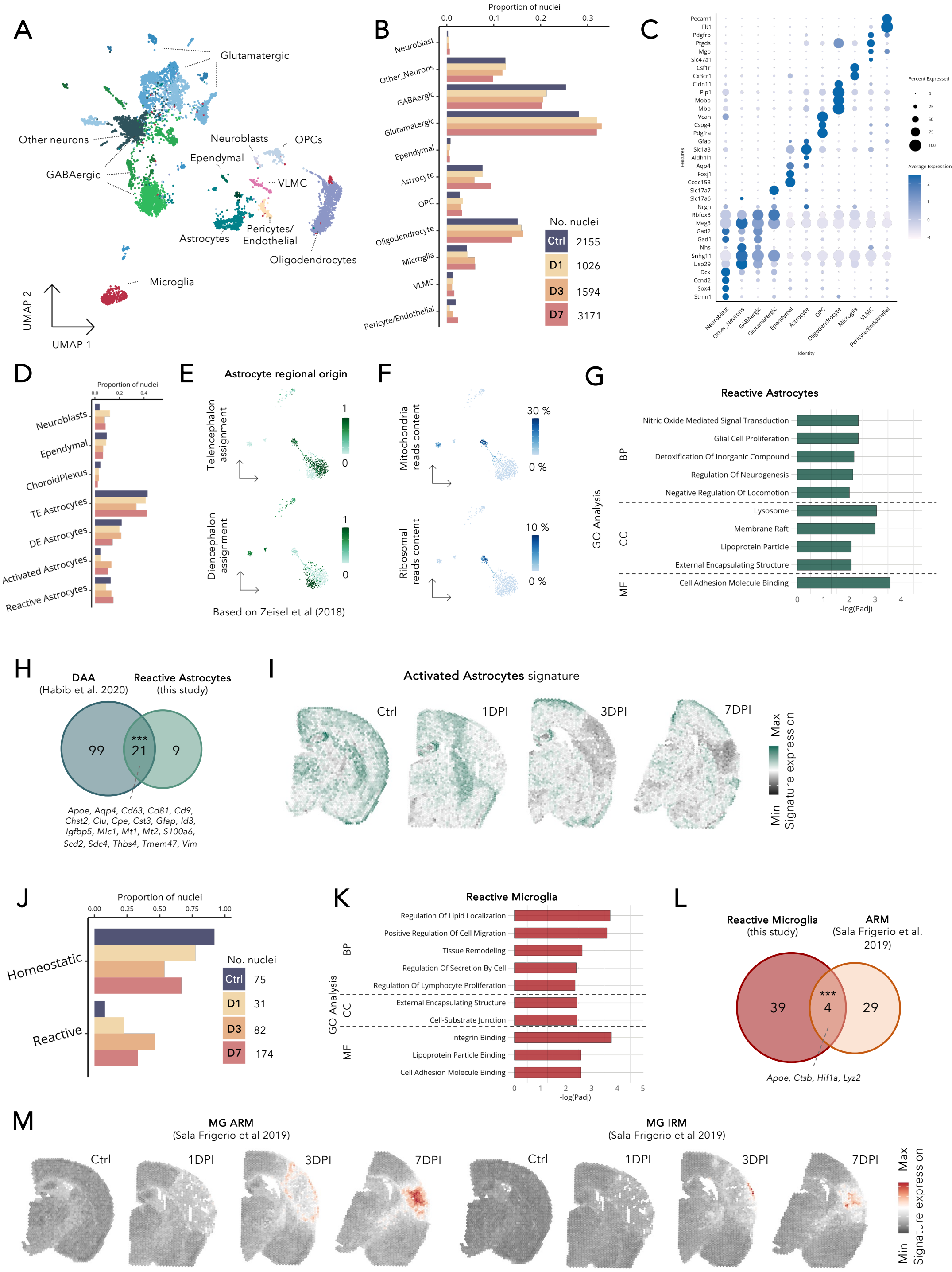

Fig. S6. Single-nucleus transcriptomic profiling of post-ischemic brain reveals rise of reactive glia populations. A) UMAP of cell populations from post-MCAO mouse brains (n = 7946). **B**) Proportions of cell populations in the individual time points. **C**) Marker genes of cell populations. Expression level (color scale) and the percentage of the population expressing the marker (dot size) are shown. **D**) Proportions of astroependymal populations in the individual time points. **E**)

Projection of the brain region origin. Mouse brain cell atlas of Zeisel et al. (19) was used for the label transfer. **F**) Percentage of mitochondrial (upper) and ribosomal (bottom) reads per cell. **G**) Representative enriched gene ontology (GO) terms from the reactive astrocyte markers. FDR-adjust *P* value is shown. **H**) Shared markers between the signatures of disease-associated (DAA, Habib et al. (20)) and this study's reactive astrocytes. Hypergeometric p-value ***P < 0.001. **I**) Projection of activated astrocytes module calculated using the top 15 population marker genes (fold-change sorted) quantified as difference in the average expression between them and randomly selected genes. **J**) Proportions of microglial populations across the timepoints. **K**) Representative enriched gene ontology (GO) terms from the reactive microglial markers. FDR-adjust *P* value is shown. **L**) Shared markers between this study's reactive microglia and activated response microglia (ARM, Sala Frigerio et al. (21)). Hypergeometric p-value ***P < 0.001. **M**) Projection of module scores for reactive microglial populations from Sala Frigerio et al.(21), characterized by 33 genes (ARM) and 41genes (IRM), respectively. Full gene lists are in Dataset S5.

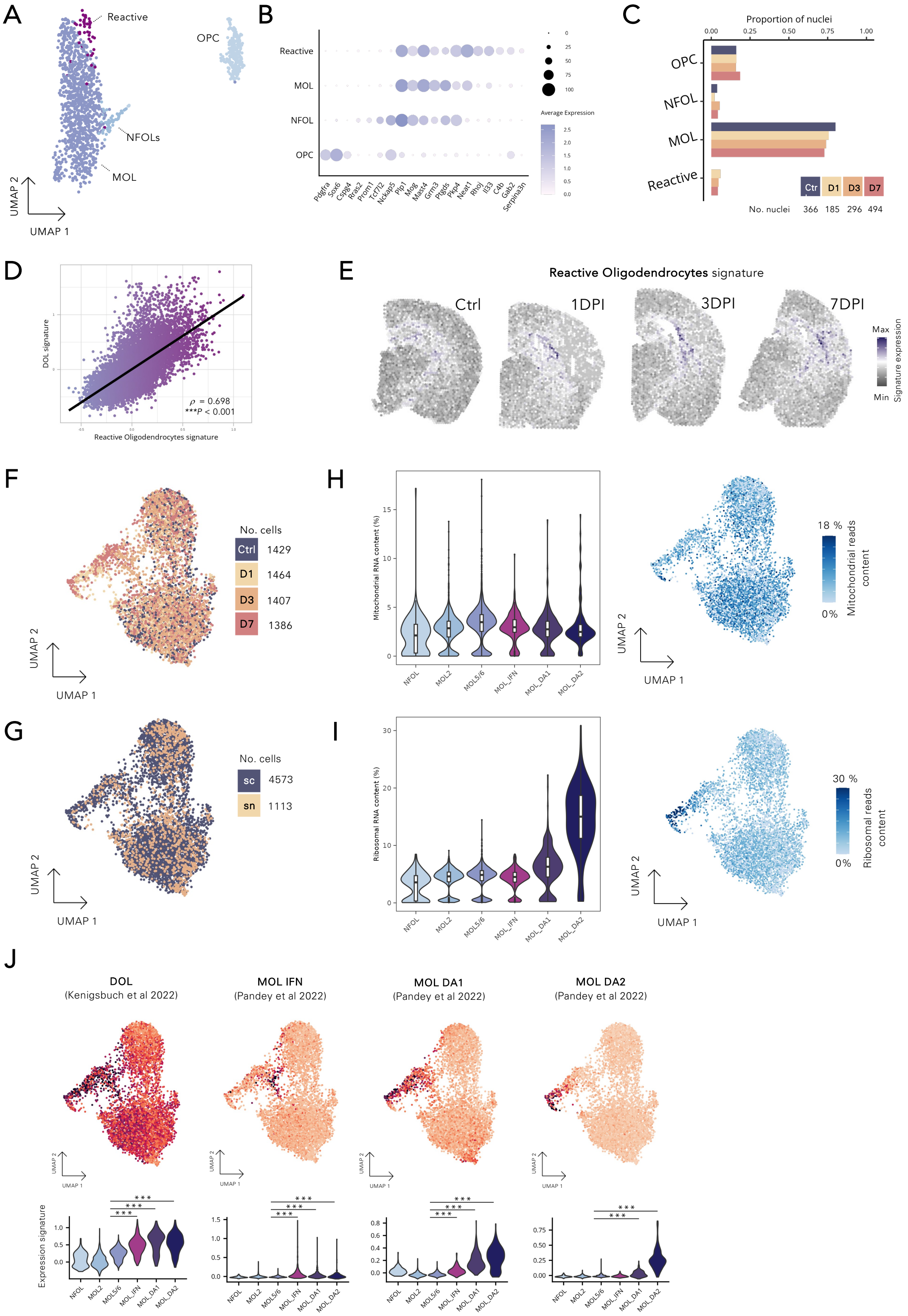
A
Reactive
OPC
NFOLs
MOL
UMAP 2
UMAP 1
B
Reactive
MOL
NFOL
OPC
Pdgfra
Sox6
Cspg4
Rras2
Prom1
Tcf7l2
Nckap5
Plp1
Mog
Mast4
Grm3
Ptgds
Pkp4
Neat1
Rhoj
Il33
C4b
Gab2
Serpina3n
Average Expression
C
Proportion of nuclei
0.00
0.25
0.50
0.75
1.00
OPC
NFOL
MOL
Reactive
Ctr
D1
D3
D7
No. nuclei
366
185
296
494
D
DOL signature
Reactive Oligodendrocytes signature
ρ = 0.698
***P < 0.001
E
Reactive Oligodendrocytes signature
Ctrl
1DPI
3DPI
7DPI
Max
Min
Signature expression
F
No. cells
Ctrl 1429
D1 1464
D3 1407
D7 1386
UMAP 2
UMAP 1
H
Mitochondrial RNA content (%)
NFOL
MOL2
MOL5/6
MOL_IFN
MOL_DA1
MOL_DA2
18 %
0%
Mitochondrial reads content
UMAP 2
UMAP 1
G
No. cells
sc 4573
sn 1113
UMAP 2
UMAP 1
I
Ribosomal RNA content (%)
NFOL
MOL2
MOL5/6
MOL_IFN
MOL_DA1
MOL_DA2
30 %
0%
Ribosomal reads content
UMAP 2
UMAP 1
J
DOL
(Kenigsbuch et al 2022)
MOL IFN
(Pandey et al 2022)
MOL DA1
(Pandey et al 2022)
MOL DA2
(Pandey et al 2022)
Expression signature
NFOL
MOL2
MOL5/6
MOL_IFN
MOL_DA1
MOL_DA2

Fig. S7. (previous page) Analysis of single-nucleus and single-cell oligodendrocyte MCAO datasets. **A**) UMAP of oligodendrocyte lineage cells (n = 1341 nuclei). Abbreviations: oligodendrocyte precursor cells, OPC; newly formed OLs, NFOL; mature OLs, MOL. **B**) Marker genes of cell populations. Expression level (color scale) across oligodendrocyte-lineage populations and the percentage of the population's cells expressing the marker (dot size) are shown. **C**) Proportions of astroependymal cell populations across the sampled timepoints. **D**) Correlation between the expression signatures of the reactive oligodendrocytes (x axis) and AD-associated disease-associated oligodendrocytes (DOL, Kenigsbuch et al.(22); y axis) in the ST dataset, measured per spot and quantified with Spearman's rank correlation coefficient ρ. **E**) Projection of reactive oligodendrocytes signature calculated using the top 15 population marker genes (fold-change sorted), quantified as difference in the average expression between them and randomly selected genes. **F**) UMAP color-labeled based on the sampled timepoints. **G**) UMAP color-labeled based on the RNA-Seq method: single-cell (sc), single-nucleus (sn). **H**) Proportion of mitochondrial read content in the oligodendrocyte populations. **I**) Proportion of ribosomal read content in the oligodendrocyte populations. **J**) Expression signatures of disease-associated populations (color scale) enriched in AD (Kenigsbuch et al.(22)) and across multiple neurodegenerative models (Pandey et al.(23)). Values are shown in the violin plots below, statistical differences assessed using Wilcoxon rank sum test with FDR-adjusted *P* values (****P* < 0.001).

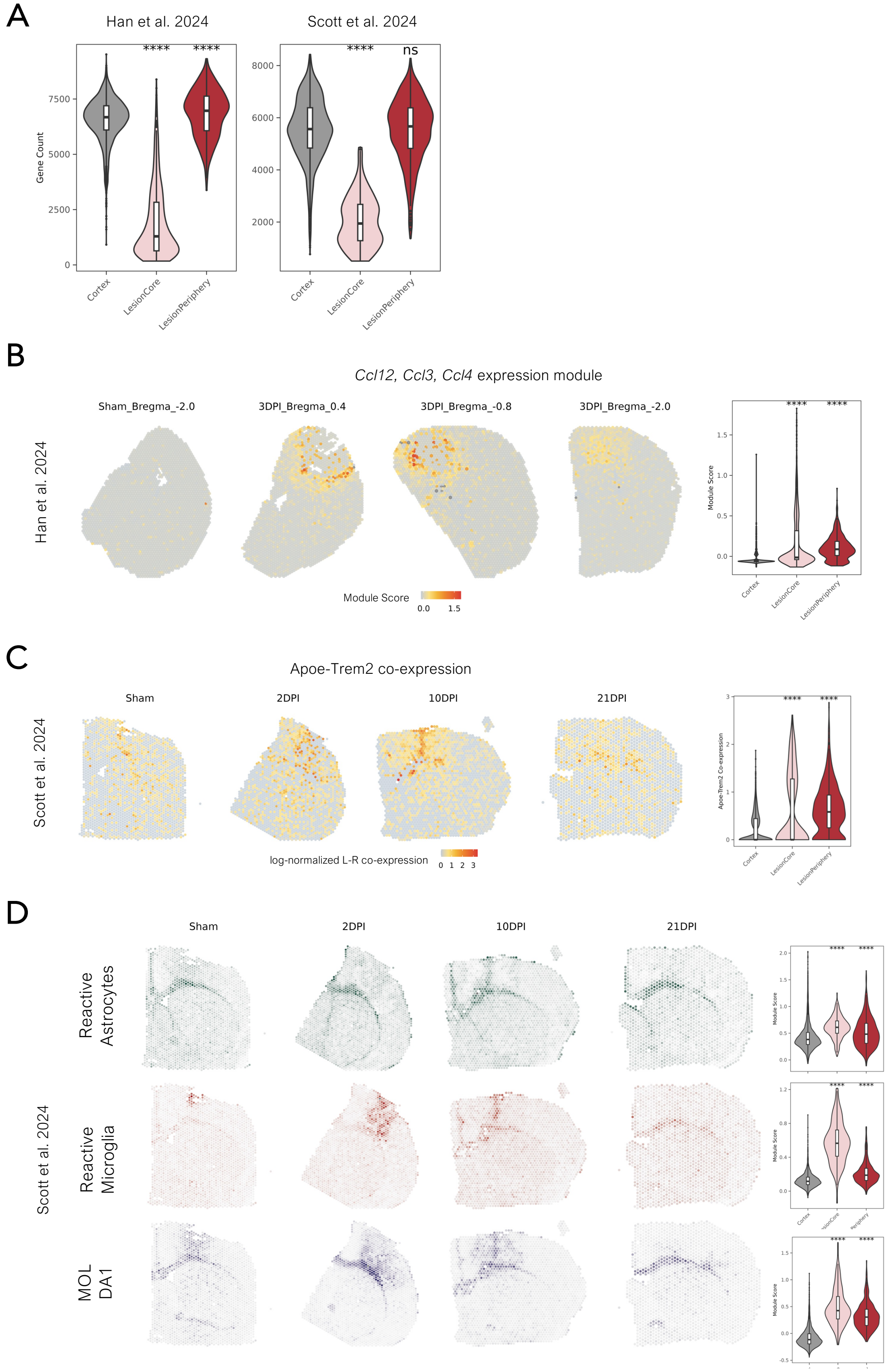
A
Han et al. 2024
Scott et al. 2024
Gene Count
Cortex
LesionCore
LesionPeriphery
ns
B
Ccl12, Ccl3, Ccl4 expression module
Sham_Bregma_-2.0
3DPI_Bregma_0.4
3DPI_Bregma_-0.8
3DPI_Bregma_-2.0
Han et al. 2024
Module Score
C
Apoe-Trem2 co-expression
Sham
2DPI
10DPI
21DPI
Scott et al. 2024
log-normalized L-R co-expression
D
Sham
2DPI
10DPI
21DPI
Reactive Astrocytes
Reactive Microglia
MOL DA1
Scott et al. 2024

Fig. S8. (previous page) Meta-analysis of publicly available ischemia-related spatial transcriptomics. A) Gene count in the areas of intact cortex and lesions. Statistical significance of difference in gene count between the intact and lesioned area was assessed by an unpaired, two-tailed t-test. **B**) Projection of module score of CC chemokine expression (*Ccl12, Ccl3, Ccl4*) per spot in selected Han et al. (8) sections. Absolute values are shown in the right-side violin plot. **C**) Spatial organization of *Apoe-Trem2* co-expression with absolute values shown in the right-side violin plot in Scott et al. (9) sections. **D**) Projections of reactive glial signatures calculated using the top 15 population marker genes (fold-change sorted) quantified as difference in the average expression between them and randomly selected genes.

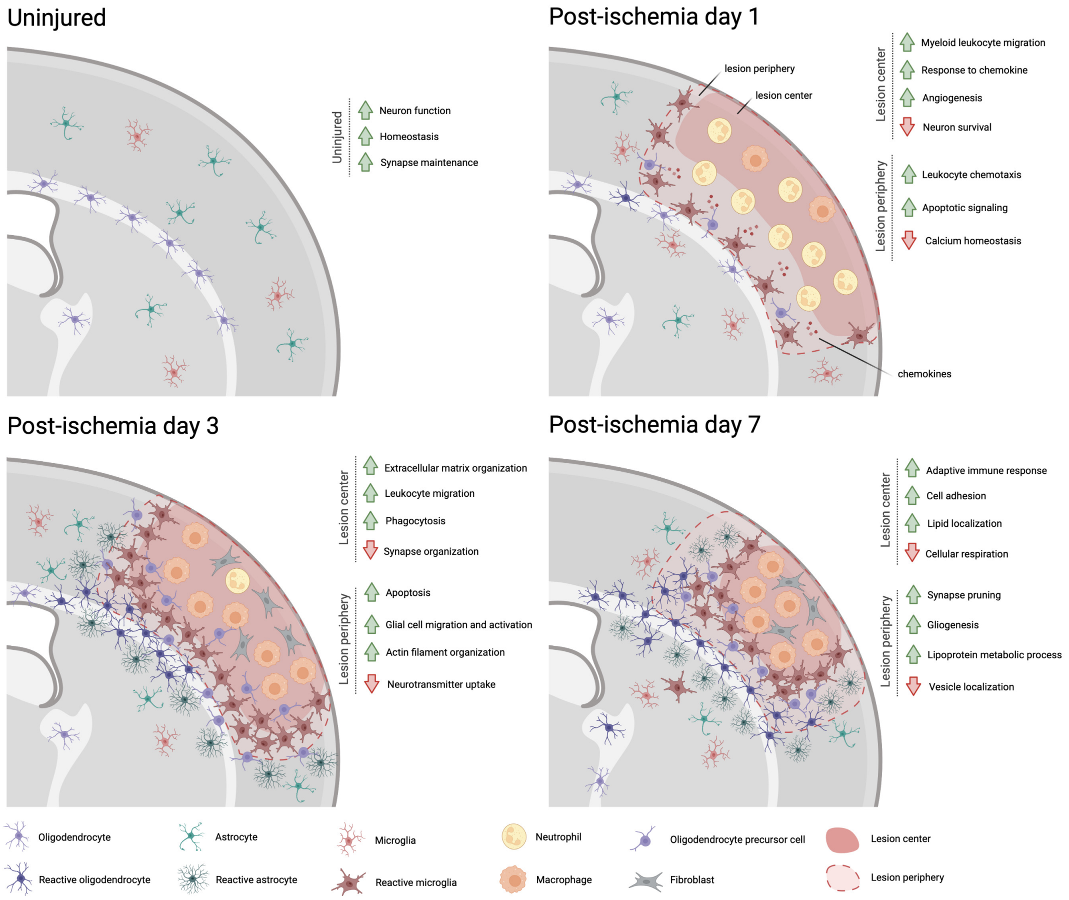

Fig. S9. Graphical summary of the study. Uninjured) In the uninjured brain, glia are scattered throughout the brain parenchyma, supporting proper neuronal function. Post-ischemia day 1) The early response is mediated by invading peripheral immune cells, coupled with a pronounced inflammatory response. **Post-ischemia day 3**) Glia migrate, proliferate, and acquire reactive states. Tissue on the lesion periphery densifies. **Post-ischemia day 7**) Peripheral immune cells are maintained within the lesion center. The glial scar matures, forming a composite barrier between the lesion center and the brain parenchyma. The reactive glial signature extends beyond the lesion, reaching white matter tracts and striatum.

Dataset S1 (separate file). Results of differential gene expression analysis for respective brain region as defined in the control section. The last tab also includes top 200 genes for respective brain regions as defined by Allen Brain Atlas *in-situ* hybridization atlas. For further details refer to *Region annotation and comparison to Allen Brain Atlas* method section.

Dataset S2 (separate file). Results of differential gene expression analysis for respective ischemic regions and their gene ontology enrichment analysis. For further details refer to *Region annotation and comparison to Allen Brain Atlas* and *Pathway enrichment* method sections.

Dataset S3 (separate file). Correlation matrix of per-spot cell type ratios and gene expression, respectively for each timepoint. For further details refer to *RCTD deconvolution* method section.

Dataset S4 (separate file). Results of spatial ligand-receptor analysis respectively for each timepoints. For further details refer to *Spatially-resolved ligand-receptor analysis with SpaTalk* method section.

Dataset S5 (separate file). Results of differential gene expression and pathway enrichment analysis for glial populations in single-nucleus (SN) and integrated single-nucleus + single-cell datasets (intSNSC). Additionally, marker lists of publicly available datasets are appended. For further details refer to *Data analysis* and *Metadata analysis* method sections.

Dataset S6 (separate file). Results of immunohistochemistry validations containing volume and colocalization values. For further details refer to *Cell counting* and *Colocalization* method sections.

SI References

1. P. Honsa, *et al.*, Generation of reactive astrocytes from NG2 cells is regulated by sonic hedgehog. *Glia* **64**, 1518–1531 (2016).
2. P. Androvic, *et al.*, Decoding the Transcriptional Response to Ischemic Stroke in Young and Aged Mouse Brain. *Cell Rep.* **31**, 107777 (2020).
3. Y. Hao, *et al.*, Integrated analysis of multimodal single-cell data. *Cell* **184**, 3573-3587.e29 (2021).
4. S. M. Sunkin, *et al.*, Allen Brain Atlas: an integrated spatio-temporal portal for exploring the central nervous system. *Nucleic Acids Res.* **41**, D996 (2013).
5. Y. Zhou, *et al.*, Metascape provides a biologist-oriented resource for the analysis of systems-level datasets. *Nat. Commun.* **10**, 1–10 (2019).
6. F. Zeng, *et al.*, Single-cell analyses reveal the dynamic functions of Itgb2+ microglia subclusters at different stages of cerebral ischemia-reperfusion injury in transient middle cerebral occlusion mice model. *Front. Immunol.* **14**, 1114663 (2023).
7. I. Korsunsky, *et al.*, Fast, sensitive and accurate integration of single-cell data with Harmony. *Nat. Methods 2019 1612* **16**, 1289–1296 (2019).
8. B. Han, *et al.*, Integrating spatial and single-cell transcriptomics to characterize the molecular and cellular architecture of the ischemic mouse brain. *Sci. Transl. Med.* **16**, 1323 (2024).
9. E. Y. Scott, *et al.*, Integrating single-cell and spatially resolved transcriptomic strategies to survey the astrocyte response to stroke in male mice. *Nat. Commun.* **15**, 1584 (2024).
10. A. M. Bolger, M. Lohse, B. Usadel, Trimmomatic: a flexible trimmer for Illumina sequence data. *Bioinformatics* **30**, 2114–2120 (2014).
11. E. Kopylova, L. Noé, H. Touzet, SortMeRNA: fast and accurate filtering of ribosomal RNAs in metatranscriptomic data. *Bioinformatics* **28**, 3211–3217 (2012).
12. A. Dobin, *et al.*, STAR: ultrafast universal RNA-seq aligner. *Bioinformatics* **29**, 15–21 (2013).
13. S. Anders, P. T. Pyl, W. Huber, HTSeq—a Python framework to work with high-throughput sequencing data. *Bioinformatics* **31**, 166–169 (2015).
14. M. I. Love, W. Huber, S. Anders, Moderated estimation of fold change and dispersion for RNA-seq data with DESeq2. *Genome Biol.* **15**, 550 (2014).
15. A. M. Newman, *et al.*, Determining cell type abundance and expression from bulk tissues with digital cytometry. *Nat. Biotechnol.* (2019) https:/doi.org/10.1038/s41587-019-0114-2.
16. A. T. L. Lun, *et al.*, EmptyDrops: distinguishing cells from empty droplets in droplet-based single-cell RNA sequencing data. *Genome Biol.* **20**, 63 (2019).
17. M. D. Young, S. Behjati, SoupX removes ambient RNA contamination from droplet-based single-cell RNA sequencing data. *Gigascience* **9** (2020).
18. D. M. Cable, *et al.*, Robust decomposition of cell type mixtures in spatial transcriptomics. *Nat. Biotechnol.* (2021) https:/doi.org/10.1038/s41587-021-00830-w.
19. A. Zeisel, *et al.*, Molecular Architecture of the Mouse Nervous System. *Cell* **174**, 999-1014.e22 (2018).
20. N. Habib, *et al.*, Disease-associated astrocytes in Alzheimer's disease and aging. *Nat. Neurosci.* **23**, 701–706 (2020).

21. C. Sala Frigerio, *et al.*, The Major Risk Factors for Alzheimer's Disease: Age, Sex, and Genes Modulate the Microglia Response to Aβ Plaques. *Cell Rep.* **27**, 1293-1306.e6 (2019).
22. M. Kenigsbuch, *et al.*, A shared disease-associated oligodendrocyte signature among multiple CNS pathologies. *Nat. Neurosci.* (2022) https:/doi.org/10.1038/s41593-022-01104-7.
23. S. Pandey, *et al.*, Disease-associated oligodendrocyte responses across neurodegenerative diseases. *Cell Rep.* **40**, 111189 (2022).
